# Integrating Epoxidation, High-Resolution Mass Spectrometry and Ultraviolet Spectroscopy to Unravel the Complex Profile of Boswellic Acids and Related Compounds in the *Boswellia serrata* Gum Resin Extract

**DOI:** 10.3390/molecules29204967

**Published:** 2024-10-21

**Authors:** Andrea Castellaneta, Ilario Losito, Stefania Cometa, Francesco Busto, Elvira De Giglio, Tommaso R. I. Cataldi

**Affiliations:** 1 Dipartimento di Chimica, Università degli Studi di Bari “Aldo Moro”, Via Orabona 4, 70126 Bari, Italy; andrea.castellaneta@uniba.it (A.C.); francesco.busto@uniba.it (F.B.); tommaso.cataldi@uniba.it (T.R.I.C.); 2 Centro Interdipartimentale SMART, Università degli Studi di Bari “Aldo Moro”, Via Orabona 4, 70126 Bari, Italy; 3 Jaber Innovation s.r.l., Via Calcutta 8, 00144 Rome, Italy; stefania.cometa@jaber.it; 4 Consorzio Interuniversitario Nazionale per la Scienza e Tecnologia dei Materiali, Via Giuseppe Giusti, 9, 50121 Florence, Italy

**Keywords:** boswellic acids, *Boswellia serrata*, epoxidation reaction, *meta*-chloroperoxybenzoic acid, UV spectroscopy, high-resolution tandem mass spectrometry

## Abstract

The chemical characterization of natural products is often a complex task that demands powerful analytical techniques. Liquid chromatography with high-resolution tandem mass spectrometry (HRMS/MS) is often employed, yet it can face hard challenges when isomeric species are present, and reference standards are lacking. In such cases, the confidence level in compound identification can be significantly improved by the collection of orthogonal information on target analytes. In this work, 23 key compounds in *Boswellia serrata* extract (BSE), 12 of which correspond to boswellic acids (BAs) and 11 to triterpenoidic acid isomers, were identified by combining RPLC followed by serial UV and ESI(-)-FTMS and FTMS/MS detections with the evaluation of the reactivity towards C=C bond epoxidation with *meta*-chloroperoxybenzoic acid (*m*-CPBA), proposed as a fast chemical tool to gather information about C=C bond steric hindrance, a key structural feature of BAs and related compounds. The interpretation of UV spectra acquired after chromatographic separation corroborated the identification of the substitution patterns of enonic and dienic residues in ketoboswellic and dehydroboswellic acids. Moreover, MS/MS based on higher-energy collision-induced dissociation (HCD) unveiled new fragmentation pathways, providing important structural details on target analytes. The integrated approach developed during this study might pave the way for a deeper understanding of the BSE bioactive properties. Moreover, it can be considered an example of a more general strategy for the analysis of complex mixtures of natural compounds including also isomeric species.

## 1. Introduction

The increasing interest in traditional medicine is driven by the potential of medicinal plants to provide sustainable and cost-effective drug candidates [1,2]. Many health practices in developing countries still rely heavily on their ancient, rich knowledge of herbal medicine [1,3]. Frankincense, also known as olibanum, is a prime example of a plant-derived product used in Ayurvedic herbal formulations to treat various human diseases [4,5].

Frankincense, derived from the French term “franc encens”, meaning pure incense, is an oleogum resin obtained from trees of the genus *Boswellia Roxb.* ex *Colebr*. in the Burseraceae family [6,7]. The resin is obtained after the coagulation, drying, and purification of the sticky and milky liquid that is secreted when the bark of the Boswellia tree is incised at the trunk and branches level [7]. Frankincense has been used as an adhesive agent and in cosmetic formulations [8], but, in ancient times, it was burned during religious ceremonies and rituals [2,6,8] to perfume worship places and to prevent the spread of contagion in these crowded areas. Indeed, both antimicrobial and antifungal properties have been recognized in frankincense smoke [9]. Moreover, the powdered gum resin and the extracts obtained from *Boswellia* spp. have been used as dietary supplements for the treatment of various inflammatory and gastrointestinal diseases [2,3,5,10,11,12,13].

The chemical composition of the *Boswellia* oleogum resin is based on the ether solubility of its components. The ether-insoluble fraction mainly consists of polysaccharides, while the ether-soluble fraction contains a large variety of terpenes and terpenoids [5,12]. Among these, boswellic acids (BAs) have been recognized as the major bioactive compounds [8,14]. BAs, first isolated by Winterstein and Stein in 1932, are a class of pentacyclic triterpenoid acids (PTAs) biosynthetically related to α and β amyrins [15,16,17]. The chemical structures and names of BAs reported in previous studies [8,17] are detailed in Table S1 of the Supplementary Materials. BAs can be grouped into two classes, namely, oleanane-type (O-type) BAs, like α-boswellic acid (α-BA) and its derivatives, and ursane-type (U-Type) BAs, like β-boswellic acid (β-BA) and its derivatives, according to the presence of the oleanane or ursane backbone in the chemical structure, respectively (see Table S1).

The interest in BAs has recently surged due to their significant role in the inhibition of inflammatory processes. 11-keto-β-boswellic acid (β-AKBA) and the 3-acetyl 9,11-dehydro-β-boswellic acid (β-AHBA) were recognized as powerful noncompetitive inhibitors of the 5-lipoxygenase, i.e., a key enzyme involved in the biosynthesis of leukotrienes [18,19]. Additionally, β-BA and β-AKBA were recognized as competitive inhibitors of human leucocyte elastase (HLE), a proteolytic enzyme involved in cartilage deterioration, released during inflammation processes [18,20]. Moreover, β-AKBA, along with incensole acetate (another component of the Boswellia gum resin), was found to inhibit the nuclear transcription factor κB (NFκB), which is involved in the induction of inflammatory processes [21,22]. Among the 25 species belonging to the genus *Boswellia*, *Boswellia serrata* (*B. serrata*) is one of the richest sources of BAs [11,23]. Its gum resin extract has been employed as a dietary supplement for the treatment of various conditions such as rhinitis, asthma, arthritis, and skin disorders [11]. 

Given the growing medical interest, various analytical methods have been developed for the characterization of BAs both in commercial products and biological fluids [10]. Gas chromatography (GC) [24,25], supercritical fluid chromatography (SFC) [10], and, most frequently, reversed-phase liquid chromatography (RPLC) [4,7,11,12,23,26,27], have been employed to separate BAs and other components in Boswellia gum resin extracts. Matrix-assisted laser desorption ionization–mass spectrometry (MALDI-MS) was recently proposed to evaluate the authenticity of commercial frankincense products [28]. However, such shotgun MS analysis is unable to separate isomeric products; thus, the hyphenation between chromatography and mass spectrometry remains the most powerful analytical tool for the identification and characterization of the isomeric forms of BAs [10]. Notably, reversed-phase liquid chromatography (RPLC) and high-resolution tandem mass spectrometry (HRMS/MS), based on a quadrupole time-of-flight instrument, have been recently employed by Katragunta et al. [4] for the characterization of BAs and other triterpenoids in *B. Serrata* oleogum resin. 

In the present study, RPLC-HRMS/MS was adopted for the characterization of BAs and their isomers (see Table S2) in the lipophilic extract of *B. Serrata* resin, using higher-energy collision dissociation (HCD) for the acquisition of more structurally informative MS/MS spectra, especially in the low *m*/*z* region. Minor but significant differences were recognized even in the HCD-HRMS/MS spectra of BA isomers exhibiting only slight structural differences, such as α-BA, β-BA, and epi-β-BA. Conversely, less informative MS/MS data were achieved in the case of 3-acetyl α-boswellic acid (α-ABA), 3-acetyl β-boswellic acid (β-ABA), and other acetylated isomers, due to the prevalence of the acetate ion in spectra. To address this issue, we explored the differential reactivity of BAs and their isomers to epoxidation with *meta*-chloroperoxybenzoic acid (*m*-CPBA), unveiling details of their C=C bond steric hindrance. Additionally, UV spectra were acquired after RPLC separation to confirm the substitution pattern of enonic and dienic chromophores in ketoboswellic and dehydroboswellic acids.

## 2. Results

### 2.1. A General Overview on BAs and Related Compounds in the Boswellia serrata Extract by RPLC-ESI(−)-FTMS

**Figure 1:**
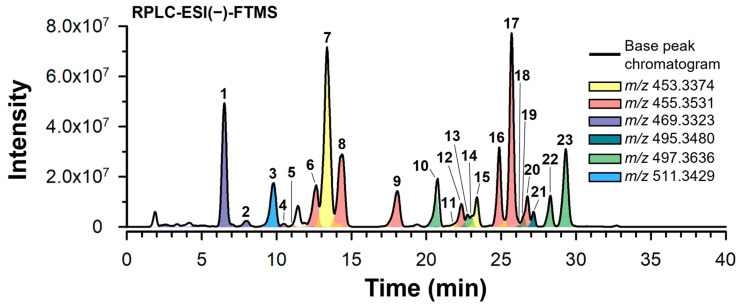
RPLC-ESI(−)-FTMS base peak chromatogram (BPC) obtained for a 100 μg/mL methanol solution of dried *Boswellia serrata* extract (straight black line). The color-shaded area traces correspond to the extracted ion chromatograms (EIC) referring to known boswellic acids (BAs) as deprotonated molecules ([M−H]^−^) (see Table S1). The EIC peaks of all the identified BAs and BA isomers (see Table 1) were numbered following the elution order.

Owing to the presence of a carboxylic acid moiety (see Table S1, Supplementary Materials), BAs can be easily converted in the corresponding deprotonated species ([M−H]^−^) by negative-ion electrospray ionization (ESI). Hence, ESI has been widely adopted as a suitable approach for the sensitive detection of BAs based on mass spectrometry [4,7,12,13,29,30,31,32]. The RPLC-ESI(−)-FTMS base peak chromatogram (BPC) obtained from a 100 μg/mL methanol solution of lyophilized *Boswellia serrata* extract (BSE) is, thus, shown in Figure 1 (black line). All major peaks in the BPC could be related to *m*/*z* values consistent with the theoretical *m*/*z* values of the [M−H]^−^ ions of known BAs (see Table S1). However, the number of peaks detected in the corresponding extracted ion chromatograms (EICs) often exceeded the number of known isomeric BAs, thus indicating the presence of additional isomeric species. In the present study, such complexity was tackled by integrating RPLC-ESI(-)-FTMS and FTMS/MS analyses with UV spectroscopy and the evaluation of C=C bond reactivity towards epoxidation. Notably, the C=C bond of all monounsaturated BAs (i.e., α-BA, β-BA) and their acetylated derivatives (α-ABA, β-ABA) exhibits a moderate (see α-BA and α-ABA) to heavy (see β-BA and β-ABA) steric hindrance. The steric accessibility of the double bond is known to affect the epoxidation reaction yield when *m*-CPBA is used as the oxidizing agent [33]. Therefore, epoxidation with *m*-CPBA was employed as a chemical tool to differentiate BAs and their isomers in terms of steric accessibility of the C-C double bond. 

Additionally, the evaluation of UV absorption properties played a major role for the unambiguous identification of BAs having conjugated dienic and enonic chromophores. The following sections will describe in detail how the epoxidation reactivity and the spectroscopic evidence were combined with the rationalization of the elution times and fragmentation patterns to tentatively identify the species responsible for the major components of BSE detected by negative-ion electrospray mass spectrometry.

### 2.2. The Role of the C=C Bond Epoxidation Reaction in Supporting the Identification of α-BA, β-BA, α-ABA, β-ABA, and Their Isomers by High-Resolution Tandem Mass Spectrometry

**Figure 2:**
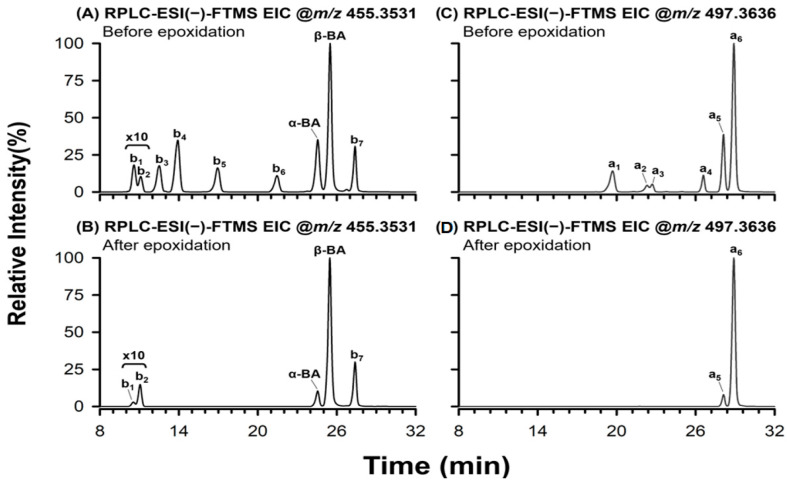
Extracted ion chromatograms (EICs) obtained by RPLC-ESI(−)-FTMS and referring to the *m*/*z* values of [M−H]^−^ ions of α-BA and β-BA (panels **A**,**B**) and their acetylated forms (panels **C**,**D**) before (panels **A**,**C**) and after (panels **B**,**D**) the epoxidation reaction with m-CPBA. An accuracy threshold of 5 ppm was set for ion current extraction. The presence of α-BA and β-BA in the *Boswellia serrata* extract was confirmed using analytical standards. The tentative assignment of the remaining EIC peaks was based on retention order, MS/MS fragmentation patterns, and reactivity towards m-CPBA (see Section 2.2 for details). Peaks b1, b2, b3, b4, b5, b6, and b7 were tentatively attributed to OA, UA, α-7,24-TDA, α-EA, β-EA, LA, and epi-β-BA (see Table S2). The observed reactivity towards the epoxidation reaction allowed the identification of peaks a5 and a6 to α-ABA and β-ABA, respectively; a1, a2, and a3 peaks were tentatively ascribed to the acetylated forms of α-7,24-TDA, α-EA, and β-EA.

β-BA is one of the most abundant BAs in the oleogum resin obtained from *B. serrata* trees [11,23]. It is classified as a U-type boswellic acid, differing from its isomer α-BA (an O-type boswellic acid) by the position of the two methyl groups on ring E (see Table S1). In this study, the presence of both β-BA and α-BA in BSE could be confirmed using suitable analytical standards. The identification was based on retention time alignment and further corroborated by the similarities observed in the FTMS/MS spectra. Consequently, peaks 16 and 17 in Figure 1 were recognized as α-BA and β-BA, respectively. Interestingly, the extraction of current related to the [M−H]^−^ ion of α-BA and β-BA (*m*/*z* 455.3531) revealed the presence of seven additional isomers, evidenced in the EIC shown in Figure 2A. As can be inferred from the comparison between Figure 2A,B, four of the nine species detected in the EIC trace (namely, those related to peaks b3, b4, b5, and b6 in Figure 2A) disappeared after the epoxidation reaction. Conversely, the limited depletion of α-BA, β-BA, and the three remaining isomers (see peaks b1, b2, and b7 in Figure 2A,B) was ascribed to the presence of a more sterically hindered C-C double bond, limiting the yield of the epoxidation reaction. Notably, α-BA was much more reactive than β-BA, since a 24% reduction was observed for the α-BA/β-BA EIC peak area ratio after the epoxidation reaction. 

It is worth noting that m-CPBA was used as an excess reagent for the epoxidation (see Section 4.2), to ensure that eventual variations in its purity (which is below 80% for commercial m-CPBA) could not influence negatively the reaction yield. Moreover, a specific test was performed by repeating the reaction on three separate aliquots of the same equimolar mixture of standard α-BA and β-BA and then calculating the ratio between the EIC peak areas of the two species after the reaction, as inferred from RPLC-ESI(-)-FTMS analysis. As a result, a 9% variability (relative standard deviation) on the average ratio between peak areas of residual α-BA and β-BA was observed, thus confirming the good repeatability of the reaction.

The higher reactivity of α-BA towards epoxidation reflected the better accessibility of the double bond at C13 since no methyl group was linked to C19 (see Table S1). Interestingly, this structural feature likely played a key role also in determining the lower retention of α-BA, compared to β-BA, on the C18 stationary phase, clearly inferred from Figure 2. Indeed, the better accessibility of the C=C bond on ring E in the case of α-BA should emphasize its negative effect on the interaction with the hydrophobic stationary phase, thus leading to a slightly lower retention time. Regarding the other peaks detected in the EICs of Figure 2A,B, a noticeable reduction was observed for the b1/β-BA EIC peak area ratio after epoxidation, whereas the b2/β-BA and b7/β-BA EIC peak ratios were not significantly affected by the epoxidation process. These findings will be further discussed later in the paper.

**Figure 3:**
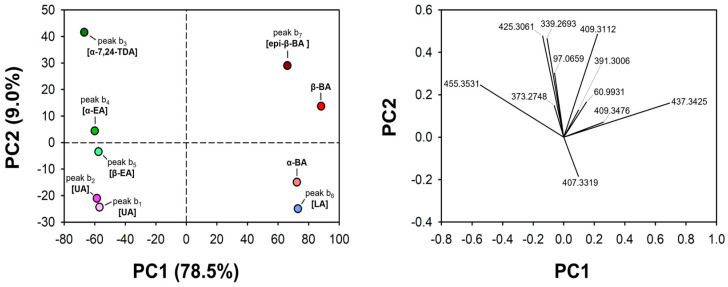
Score (**left**) and loading (**right**) plots showing the graphical output of the principal component analysis (PCA) performed on ESI(−)-FTMS/MS data acquired for α-BA, β-BA, and the further 7 isomers (i.e., OA, UA, α-7,24-TDA, α-EA, β-EA, LA, and epi-β-BA) tentatively identified for the same *m*/*z* ratio in the Boswellia serrata extract (see the main text for details). The fragment *m*/*z* ratios were used as the variables, with the intensities of the corresponding signals representing their values. For the sake of simplicity, only variables having loading values higher than 0.2 were displayed in the loading plot. Each point in the score plot was labelled following the EIC peak nomenclature shown in Figure 2A. The corresponding putative attributions are shown in square brackets.

As inferred from Figures S1 and S2 (Supplementary Materials), α-BA, β-BA, and their isomers exhibited a variable level of similarity in the FTMS/MS spectra of the corresponding [M−H]^−^ ions. This feature was highlighted also by the outcome of the principal component analysis (PCA) performed on FTMS/MS data, shown in Figure 3. Specifically, the theoretical *m*/*z* ratios of the fragment ions, calculated from the chemical formulas hypothesized from experimental ratios, were set as the PCA variables, with the corresponding spectral intensities representing their numerical values. Only peak signals with an intensity higher than 2% of the base peak were considered for variable selection (see Section 4.5 for details). The objects for PCA corresponded to the isomeric species, including α-BA and β-BA; the data matrix was centered, and no further data pretreatment was applied. As a result, two sharply separated groups of chemical species were observed along the PC1 axis in the score plot shown in Figure 3, the separation being consistent with the elution order observed in Figure 2A. In fact, all early eluting species (b1, b2, b3, b4, and b5) exhibited negative PC1 values, while species eluting later (b6, α-BA, β-BA, and b7) showed positive PC1 values. The PCA score plot indicated a similarity between isomer b7 and β-BA, confirmed by the respective FTMS/MS spectra (see Figure S1B,C). Likewise, points related to α-BA and species b6 were also close in the PCA score plot, mainly due to the very low intensity, or even the absence, of peaks compatible with the exact *m*/*z* 409.3112 (see the red-starred inset in Figure S1A,D). Additionally, the product ion compatible with an exact *m*/*z* value 407.2956 was missing in the case of isomer b6 (see the green-starred inset in Figure S1D). The PCA results served as a starting point to explore the structural similarities between α-BA and the b6 isomer, and between β-BA and the b7 one, and then to identify the cited product ions. Firstly, hydrogen/deuterium (H/D) exchange experiments (see Section 4.4) were performed to investigate the fragmentation behavior of [M−H]^−^ ions originating from α-BA and β-BA analytical standards. Indeed, both α-BA and β-BA have two exchangeable hydrogens (those involved in the carboxylic and hydroxylic OH groups); due to its higher acidity, the deuterium introduced in the carboxylic OH group is expected to be lost when deprotonated species are formed in the ESI source.

**Figure 4:**
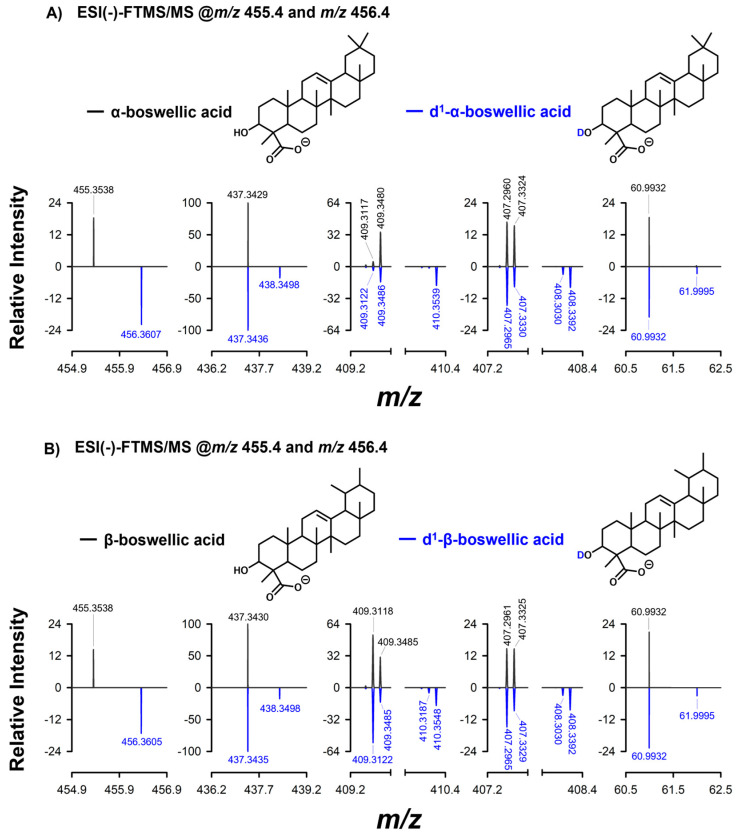
Comparison between selected magnified regions of the ESI(−)-FTMS/MS spectra of [M−H]^−^ ions of α-BA (panel **A**) and β-BA (panel **B**). The MS/MS spectra of deuterated (d1) ions are shown in blue, while those of nondeuterated [M−H]^−^ ions are shown in black.

However, the deuterium exchanged on the other OH group is stable, allowing the detection of mono-deuterated derivatives of standard α-BA and β-BA as negative ions after H/D exchange, which were then fragmented. Insets of the HCD-FTMS/MS spectra obtained for the two species, deuterated (blue lines) or not (black lines), can, thus, be compared in Figure 4A,B. The peak signals related to the precursor ions (*m*/*z* 455.3538 vs. 456.3605/456.3607) clearly showed the presence of a D atom in the molecular structure after H/D exchange. The major fragmentation event observed in the HCD-FTMS/MS spectra of nondeuterated α-BA and β-BA [M−H]^−^ ions was the loss of a water molecule (see plots A and B of Figure S1). Therefore, peaks consistent with the loss of either HDO (leading to a product ion with exact *m*/*z* 437.3425) or an H_2_O molecule (leading to a product ion with exact *m*/*z* 438.3488) were observed when the mono-deuterated (d1) forms of α-BA and β-BA were fragmented (see the second inset in plots A and B of Figure 4). This evidence partially supports the hypothesis proposed by Katragunta et al. [4] about the formation of a double bond between C2 and C3 induced by a water loss involving the OH group, occurring through a charge-remote elimination mechanism. This mechanism would explain the loss of HDO from mono-deuterated α-BA and β-BA, as the hydroxylic group on C3 is an OD group and can be lost together with an H atom linked to C2. However, an H_2_O loss was also observed from mono-deuterated α-BA and β-BA; thus, a more complex fragmentation pathway was hypothesized and is depicted in Scheme S1. First, the loss of HDO was proposed to occur also after the opening of ring A through a pericyclic rearrangement followed by tautomerism of the resulting enol, leading to a stable α,β-unsaturated carboxylic acid (see Route 1 in Scheme S1). On the other hand, the loss of H_2_O from deuterated α-BA and β-BA was tentatively explained by the deuterium transfer process shown in route 2 of Scheme S1. Here, the hydroxylic deuterium atom is initially removed by the O atom of the carboxylic group, causing the opening of ring A. Thereafter, a terminal alkyne is generated by the charge-remote loss of a nondeuterated water molecule from the enol residue. The loss of a formic acid molecule (HCOOH), followed by a further loss of H_2_ (see signals compatible with exact *m*/*z* 409.3478 and 407.3319, respectively, in Figure S1), was also a distinctive trait of the ESI(−)-FTMS/MS spectra of α-BA, β-BA and the isomeric species b6 and b7. As shown in the third inset of Figure 4A,B, the loss of HCOOH or HCOOD from mono-deuterated α-BA and β-BA ions occurred to a similar extent (see peaks compatible with an exact *m*/*z* 409.3476 and 410.3539 in blue spectra). This is tentatively explained in Scheme S1. The deuterium atom can be transferred from the hydroxylic group to the carboxylate moiety before the elimination of HCOOD, which would generate a resonance-stabilized enolate (see the top part of Scheme S1). On the other hand, the already discussed Route 2 can lead to the loss of an HCOOH molecule even in the case of monodeuterated α-BA and β-BA.

Another common peak signal for α-BA and β-BA and species b6 and b7 was detected at *m*/*z* 60.9932 (see Figure S1), a value corresponding to the hydrogen carbonate ion (HCO_3_^−^). Plots A and B of Figure 4 (see the blue line in the last inset of each panel) show that both HCO_3_^−^ (exact *m*/*z* 60.9931) and DCO_3_^−^ (exact *m*/*z* 61.9999) were detected when mono-deuterated α-BA and β-BA ions were fragmented. As shown by Route 3 in Scheme S1, the release of the deuterium carbonate ion might occur through a retro-[2+2] cycloaddition (see the fuchsia arrows) occurring on an isomeric form of the precursor ion including a 4-term cycle. The release of hydrogen carbonate would likely require a more complex electron rearrangement (see the orange arrows in Route 3), determining the migration of the deuterium atom from the hydroxylic group to the carbon skeleton of the resulting neutral fragment. The results of the H/D exchange described in Figure 4 suggest that the retro-[2+2] cycloaddition pathway occurred to a negligible extent since nondeuterated hydrogen carbonate ions were mainly generated by deuterated precursor ions of α-BA and β-BA.

The fragmentations discussed so far, i.e., the losses of water and formic acid and the release of hydrogen carbonate, can be considered the fingerprint for ring A in BA molecules. Notably, ring A in α-BA and β-BA is analogous to the first of the five condensed rings found in the lupeolic acid (LA) molecule, depicted in Table S2. The presence of peak signals related to those fragmentations in the HCD-FTMS/MS spectrum of species b6 (see Figure S1) suggests that LA could be a candidate for this isomer of α-BA and β-BA. The presence of a single and more sterically accessible C-C double bond in the case of LA (see Table S2) is also consistent with the absence of the b6 peak in the EIC trace acquired after the epoxidation with m-CPBA (see Figure 2A,B). Moreover, the presence of LA in the resinous exudate of Boswellia trees has been reported in previous studies [7,8]. Finally, the elution order observed for b6, α-BA, and β-BA in Figure 2A aligns with the one reported by Schmiech et al. [7] for LA, α-BA, and β-BA using similar chromatographic conditions. Therefore, peak b6 shown in Figure 2A was putatively recognized as LA.

Peak signals with *m*/*z* ratios compatible with exact values 409.3112, 407.2956, and 219.1391 were detected in the MS/MS spectra of α-BA and β-BA (see Figure S1A,B), but not in the case of LA (see Figure S1D). This suggested that rings C, D, and E (i.e., the discriminating part between LA and BAs structures) were involved in those fragmentations. The formation of the *m/z* 407.2956 ion was related to the loss of an ethane molecule from the [M−H−H_2_O]^−^ ions, determining the generation of a conjugated diene at the C-ring level (see the red routes in Scheme S2). The generation of the ion at *m*/*z* 409.3112 was compatible with the loss of an ethylene molecule from the E ring of [M−H−H_2_O]^−^ ions (see the blue routes in Scheme S2), leading to a 4-membered ring. The higher intensity of the *m/z* 409.3112 peak for β-BA, compared to α-BA (see Figure S1A,B), confirmed that the E ring (i.e., the structural unit where the difference between α-BA and β-BA occurs) was involved in the fragmentation event. As can be inferred from the comparison of Scheme S2A,B, a more sterically hindered product is generated in the case of α-BA, due to the geminal position of the two methyl groups on the cyclobutane ring. This feature was invoked to explain why this fragmentation route occurred to a lower extent in the case of α-BA. Finally, the product ion with *m*/*z* 219.1391 was found to be diagnostic for the presence of the C-C double bond between C12 and C13 in α-BA and β-BA. This fragment ion can be generated by a retro Diels–Alder reaction starting from [M−H−H_2_O]^−^ ions and involving the ring including that double bond (Scheme S3A).

It is worth noting that peak signals compatible with exact *m*/*z* values 409.3112, 407.2956, and 219.1391 were detected also in the HCD-FTMS/MS spectrum obtained for the b7 isomer (see Figure S1C). Moreover, the b7/β-BA EIC peak ratio remained substantially unaltered after epoxidation. As previously mentioned, the MS/MS spectrum of the b7 isomer was extremely similar to the one observed for β-BA [M−H]^−^ ions, except for the slightly higher intensity of the peak at *m*/*z* 60.9931. Since the OH and COOH residues are strictly involved in the fragmentation pathway leading to that ion (see Scheme S1), this slight difference in the fragmentation behavior was putatively attributed to the different orientation of the OH group in the C3 epimer of β-BA, namely, epi-β-BA (see Table S1), that was, thus, the identity assigned to isomer b7. The presence of epi-β-BA in Boswellia carterii was previously reported [17,34]. Notably, β-BA eluted earlier than the tentatively identified epi-β-BA (see Figure 2A), thus suggesting a higher chemical affinity of epi-β-BA towards the C18 stationary phase. This was explained with the orientation of the two polar groups (i.e., the hydroxylic and carboxylic ones) on the same side of the condensed polycyclic system.

The identification of species isomeric with α-BA and β-BA was subsequently focused on the isomers characterized by a negative PC1 coordinate in the score plot displayed in Figure 3. Among them, b3, b4, and b5 isomers were quantitatively depleted by the reaction with m-CPBA, whereas a remarkable reduction was observed for the b1/β-BA peak area ratio and the b2/β-BA ratio remained essentially unaltered (see Figure 2A,B). The ESI(−)-FTMS/MS spectra related to b1 and b2 peaks (see plots A and B of Figure S2, respectively) indicated the generation of a single relevant product ion, at *m*/*z* 407.3325. This value was compatible with the neutral loss of CH_4_O_2_, a feature known to be distinctive for the collision-induced dissociation of [M−H]^−^ ions of oleanolic (OA) and ursolic (UA) acids [35] (see Table S2). The moderate to heavy steric hindrance of the C-C double bond would explain the lower reaction yields towards m-CPBA observed for isomers b1 and b2. Based on their different relative reactivity, b1 and b2 EIC peaks were tentatively attributed to OA and UA, respectively. Indeed, the C=C bond of OA is more accessible compared to the UA one. The ESI(−)-FTMS/MS spectra averaged under b3, b4, and b5 peaks (see Figure S2) exhibited strong similarities in the high *m*/*z* region, with main peaks detected at *m*/*z* ratios compatible with/identical to exact values 425.3069, 373.2752, and 339.2696, that were previously attributed to 3-hydroxytirucallic acid isomers, such as α-EA, β-EA, and α-7,24-TDA [4] (see Table S2). In the present work, the formation of the *m*/*z* 425.3069 ion was tentatively attributed to the loss of ethane (C_2_H_6_) from α-EA, β-EA, and α-7,24-TDA [M−H]^−^ ions, leading to an extension of the double bond conjugation in the condensed polycyclic system (Scheme S3B). The formation of the *m*/*z* 373.2752 ion, implying the loss of a C_6_H_10_ neutral fragment, was ascribed to a McLafferty-type rearrangement involving the carboxylic group located in the side chain [4] (see Scheme S3C). Notably, that side chain is a common structural feature for species α-EA, β-EA, and α-7,24-TDA (see Table S2).

Interestingly, the presence of OA, UA, α-7,24-TDA, α-EA, and β-EA was assessed by Asteggiano et al. [36] in the resinous exudate of Protium heptaphyllum (Aubl.) Marchand (PH) tree. The study showed that, when separated on a C18 column, OA and UA eluted before α-7,24-TDA, α-EA, and β-EA. Moreover, 3-oxo-tirucallic acid (3-oxo-TA) eluted between β-EA and α-7,24-TDA. As discussed in the next section, the presence 3-oxo-TA was verified in BSE. Therefore, comparing the elution patterns reported by Asteggiano et al. [36] with the EIC traces shown in Figure 2A and Figure S4C, b3, b4, and b5 EIC peaks were tentatively attributed to α-7,24-TDA, α-EA, and β-EA. However, due to the lack of suitable analytical standards, the exact correspondence between each of these species and the three isomers could not be assessed.

The evaluation of the reactivity towards epoxidation with m-CPBA provided valuable structural information, especially for species occurring in BSE that exhibited poorly informative ESI(−)-FTMS/MS, like acetylated BAs. As shown in Figure 2C, six signals were recognized in the EIC trace related to the [M−H]^−^ ion of α-ABA and β-ABA (see Table S1). However, only peaks a5 and a6 were still recognizable after the epoxidation reaction (see Figure 2D). The significant reduction in the a5/a6 EIC area ratio, from 0.35 to 0.07, after the epoxidation reaction was comparable to the one observed for the α-BA/β-BA EIC area ratio. This suggested that the a5 and a6 could be attributed to the acetylated forms of α-BA and β-BA, respectively. Figure S3B,C show the ESI(−)-FTMS/MS spectra referring to peaks a5 and a6, both dominated by a single intense peak at *m*/*z* 59.0138, clearly related to the acetate anion (exact *m*/*z* 59.0139). The prevalence of this peak in the case of α-ABA and β-ABA can be explained by the proximity of the acetylated hydroxyl group to the carboxylate moiety, facilitating either the nucleophilic attack by the carboxylate negatively charged oxygen atom on C3, or a β-elimination reaction triggered by the abstraction of a proton in C2. In both cases, the acetate ion is expelled as the leaving group (see Scheme S4A). A similar behavior was hypothesized for ALA, the acetylated form of lupeolic acid (see Table S2), which is expected to exhibit a lower affinity for the C18 stationary phase compared to α-ABA and β-ABA [7]. As shown in Figure S3A, the ESI(−)-FTMS/MS spectrum for peak a4 matched those related to peaks a5 and a6. Moreover, the absence of a4 EIC after epoxidation (see Figure 2D) indicated a high reactivity, consistently with the steric availability of the C-C double bond in ALA. Thus, peaks a4, a5, and a6 were recognized as ALA, α-ABA, and β-ABA, respectively.

The acetate ion signal was detected also in the ESI(−)-FTMS/MS spectra related to a1, a2, and a3 peaks, but in these cases they were accompanied by other informative signals (see Figure S3D,E,F). Specifically, peak signals with *m*/*z* ratios compatible with or equal to exact values 467.3167 and 415.2856 indicated the same distinctive neutral losses (C_2_H_6_ and C_6_H_10_) inferred from the fragmentation of [M−H]^−^ ions of α-7,24-TDA, α-EA, and β-EA (see Scheme S3B,C). Additionally, the intense signal at *m*/*z* 437.3426 was consistent with the loss of an acetic acid molecule (AcOH, C_2_H_4_O_2_), compatible with the chemical structure of the acetylated form of those isomers of 3-hydroxytirucallic acid. As depicted in Scheme S4B, the detachment of AcOH can be induced by a McLafferty-type rearrangement. The resulting ion can undergo the loss of C_2_H_6_ from the polycyclic structure (*m*/*z* 407.2956) and the loss of C_6_H_10_ from the side chain (*m*/*z* 355.2643). As for the corresponding nonacetylated forms, the high steric accessibility of the double bond at the side chain level is in excellent accordance with the absence of a1, a2, and a3 peaks in the EIC trace acquired after the epoxidation reaction, indicating a quantitative reaction. Hence, a1, a2, and a3 peaks were tentatively attributed to α-7,24-ATDA, α-AEA, and β-AEA (see Table S2). Also in this case the lack of suitable analytical standards prevented the definitive assignment of each peak to its respective species.

A summary of species identified in BSE during the present investigation (see also Section 2.3), along with information on MS and UV features and the reactivity towards m-CPBA, is reported in Table 1.

### 2.3. Combination of UV Spectroscopy and High-Resolution Tandem Mass Spectrometry for the Unambiguous Identification of Conjugated Keto- and Dehydro-Boswellic Acids

Along with α-BA, β-BA, and their acetylated forms, 11-keto-β-boswellic acid (β-KBA) and 3-acetyl-9,11-dehydro-β-boswellic acid (β-AKBA) represent the major BAs that can act as inhibitors of enzymes involved in inflammation [3]. In particular, β-KBA and β-AKBA were recognized as powerful noncompetitive inhibitors of 5-lipoxygenase, i.e., a key enzyme for the biosynthesis of leukotrienes [7,13,23]. β-KBA is the most polar among BAs. It has been demonstrated that β-KBA is preferentially released in aqueous environments when BSE is incorporated into inorganic carriers [32,37]. When the BSE was analyzed, the extraction of the ion current for the β-KBA [M−H]^−^ ion (*m*/*z* 469.3323) resulted in the EIC trace shown in Figure S4A, in which two early eluting peaks (k1 and k2) were detected. Note that the structure of β-KBA is characterized by the presence of an enone functional group on ring C (see Table S1) and unsubstituted enones are known to exhibit a distinctive UV absorption at 215 nm [38]. A red-shift of the UV absorption band is observed when the α and β carbons are alkylated and/or the C-C double bond acts as an exocyclic unsaturation [38].

In general, the theoretical UV absorption wavelength of dienes and enones can be empirically predicted based on Woodward–Fieser’s (WF) rules [38]. The application of these rules to β-KBA resulted in a predicted absorption band at 244 nm. Detection based on UV-DAD following RPLC separation was, thus, exploited to obtain useful structural information on further species occurring in the BSE. Firstly, an excellent alignment was observed between the k1 and k2 peaks in the EIC trace of β-KBA [M−H]− ion and the first two peaks (marked by blue asterisks) detected in the RPLC-UV chromatogram acquired at 249 nm (see plots A and B in Figure S4). This wavelength matched with that of the maximum absorption observed in the UV spectra obtained for k1 and k2 peaks and was consistent with the value predicted by the WF rules. The third peak in the RPLC-UV trace at 249 nm (marked with a pink asterisk) was aligned with the k3 peak detected in the EIC trace for the *m*/*z* value 511.3429, related to the [M−H]^−^ ion of β-AKBA (see the pink trace in Figure S4A). This suggested the presence of the same functionalized enone in the structure of species related to peaks k1, k2, and k3 in Figure S4A. Additional structural information was retrieved from the ESI(−)-FTMS/MS spectrum of the k1 peak (see Figure S5A), i.e., the most intense peak observed in the EIC trace for *m*/*z* 469.3323. The formation of a hydrogen carbonate ion (*m*/*z* 60.9931), inferred from the spectrum, was diagnostic for the distinctive structure of the A-ring in BA molecules (see Scheme S1). Unlike α-BA and β-BA, the negative charge in KBA [M−H]^−^ ions can be transferred to the enone group and the resulting release of carbonic acid and methane, leading to a product ion with exact *m*/*z* 391.3006 (see Scheme S5A), was proposed to explain the presence of an intense peak at *m*/*z* 391.3009 in the MS/MS spectrum of peak k1. The MS/MS spectrum for peak k2 was similar to the one acquired for the k1 isomer, whereas the spectrum related to the k3 peak exhibited only an intense peak at *m*/*z* 59.0138. As discussed in Section 2.2, the dominant formation of the acetate ion (exact *m*/*z* 59.0139) is a common feature in the MS/MS spectra of acetylated BA [M−H]^−^. Considering the already reported abundance of β-KBA and its acetylated form in the resinous exudate of Boswellia serrata trees [3,11], the k1 and k3 peaks (the most intense peaks found in the EIC traces shown in Figure S4A) were attributed to β-KBA and β-AKBA, respectively. The similarities observed between the UV and MS/MS spectra obtained for peaks k1 and k2 suggest that the latter could correspond either to α-KBA or to the C3 epimer of β-KBA (epi-β-KBA). The presence of α-KBA as a minor constituent of the Boswellia serrata gum resin was confirmed in the work by Schmiech et al. [12]. Conversely, there is no evidence about the isolation of epi-β-KBA or its biosynthetic precursor, namely, epi-α-BA [17].

Considering additional species occurring in the BSE, peak n.7 is the second most intense peak in the base peak chromatogram shown in Figure 1. The corresponding full-FTMS spectrum revealed an intense signal at *m*/*z* 453.3377, a value compatible with the theoretical *m*/*z* (453.3374) of known dehydroboswellic acids, namely, α-DHBA and β-DHBA (see Table S1). The extraction of the corresponding ion current resulted in the green EIC trace shown in Figure S4C, dominated by a peak labeled as d1, accompanied by three less intense peaks eluting between 20 and 30 min. The structure of both α-DHBA and β-DHBA is characterized by the presence of a distinctive UV chromophore, i.e., a conjugated diene in ring C. A theoretical UV absorption maximum at 281 nm was calculated for this structural feature by the application of the WF rules [38]. However, no significant absorption was detected at this wavelength in the UV spectrum averaged for the d1 peak, as clearly indicated by the RPLC-UV/DAD trace acquired at 281 nm (see Figure S4D). Conversely, intense absorption at 281 nm was observed in the UV spectra of d2 and d3 peaks (see also the peak alignment between the EIC and UV RPLC traces in Figure S4C,D). Further insights were gained from the ESI(−)-FTMS/MS spectra of the d1 and d3 isomers (see plots C and D of Figure S5). The spectrum related to d1 was characterized by peak signals at *m*/*z* 423.2908 and 371.2593, implying, respectively, losses of C_2_H_6_ and C_6_H_10_, analogous to those observed for tirucallic acid-type derivatives (see Section 2.2). This evidence supported the identification of the d1 peak to 3-oxo-TA (see Table S2), a species lacking UV chromophores different from nonconjugated carbonyls, which explains the absence of UV absorption at 281 nm. The presence of 3-oxo-TA in *Boswellia serrata* gum resin has been already reported [4] and the elution pattern of α-7,24-TDA, α-EA, β-EA, and 3-oxo-TA was also consistent with the one observed by Asteggiano et al. [36].

In addition to absorbing at 281 nm, the d3 isomer exhibited a signal at *m/z* 60.9931 in its MS/MS spectrum (see Figure S5C), by analogy with all BA [M−H]^−^ ions. Two other peak signals were detected at *m*/*z* 359.2747 and 375.3059, values consistent with those of ions containing only C and H atoms, namely, [C_27_H_35_]^−^ and [C_28_H_39_]^−^. Unlike other BA molecules, dehydroboswellic acids include a potentially alternative ionization site corresponding to C18, since its deprotonation can lead to a resonance-stabilized carbanion. The loss of carbonic acid and up to two methane molecules can then occur, resulting in the generation of two extensively resonance-stabilized carbanions (Scheme S5B). A similar fragmentation behavior was inferred from the MS/MS spectrum of the d2 isomer. Therefore, d2 and d3 were tentatively identified as α-DHBA and β-DHBA, although the unambiguous identification of the respective peaks was prevented by the lack of suitable analytical standards.

The last two peaks shown in the RPLC-UV trace of Figure S4D, marked by orange asterisks, aligned perfectly with the d4 and d5 peaks detected in the orange-colored EIC trace of Figure S4C, referred to [M−H]^−^ ions of acetylated forms of α-DHBA and β-DHBA, i.e., α-ADHBA and β-ADHBA (*m*/*z* 495.3480). Moreover, the MS/MS spectrum acquired for d4 and d5 exhibited the presence of a single and intense peak at *m*/*z* 59.0137, typical of acetylated BAs. Peaks d4 and d5 could, thus, be attributed to α-ADHBA and β-ADHBA, although it was not possible to assign a specific identity to each of the two peaks.

## 3. Discussion

The comprehensive characterization of the boswellic acids (BAs) profile in a commercial sample of B. serrata extract (BSE) could be achieved through the synergy of different analytical approaches employed in conjunction with RPLC, including ESI(-)-FTMS and FTMS/MS analysis supported by H/D exchange experiments and PCA-based processing of data, UV-DAD detection, and the careful evaluation of the outcome of a sterically sensitive epoxidation reaction targeting the C=C bond. This multifaceted strategy enabled the identification of up to 23 species responsible for the majority of signals detected in the RPLC-ESI(−)-FTMS base peak chromatogram obtained for the BSE. Among them, 12 species were recognized as BAs, while the remaining 11 were identified as triterpenoidic acid isomers. Key findings included the varying reactivity of BAs towards the epoxidation of the C=C bond with m-CPBA, with α-BA being more reactive than β-BA. This result was consistent with the more favorable exposure of the α-BA C=C bond to the oxidizing agent, determined by the geminal position of two methyl groups close to the C=C bond, as opposed to their vicinal position in β-BA. This information was crucial also to discriminate the corresponding acetylated forms, namely, α-ABA and β-ABA, that could not be distinguished through FTMS/MS and UV analyses. The extent of the epoxidation reaction also played an important role in the identification of oleanolic and ursolic acids, as well as lupeolic acid and 3-hydroxy tirucallic acid derivatives. Additionally, BAs including conjugated double bonds in the form of enones (ketoboswellic acids) and dienes (dehydroboswellic acids) in their structures could be characterized through a careful interpretation of their UV spectra, collected after RPLC separation.

The results of this study underscore the power of an integrated analytical strategy, implementing specialized reactions to probe the steric accessibility of specific structural features, along with consolidated detection strategies following chromatographic separation. This approach can be considered particularly valuable for characterizing complex mixtures of natural compounds, especially when only a limited number of analytical standards is available. Indeed, information initially retrieved from standards (like UV spectral features and MS/MS-related fragmentation pathways) can help in understanding the behavior of further analytes that share some structural characteristics with them but whose identity cannot be assessed through comparison with the respective standard.

From a more general perspective, the described approach might be very useful to extend the knowledge of compounds occurring in complex natural products, contributing to enhancing the understanding of their properties.

## 4. Materials and Methods

### 4.1. Chemicals

Water, methanol (LC-MS grade), and chloroform (HPLC grade), used for sample preparation and RPLC-ESI-FTMS analysis, ammonium acetate and the 28% *v*/*v* ammonia solution (reagent grade), used as mobile phase additive, and meta-chloroperoxybenzoic acid (m-CPBA, reagent grade), used as epoxidating reactant, were purchased from Merck (Milan, Italy). α-boswellic acid and β-boswellic acid analytical standards were purchased from Merck (Milan, Italy). The powder extract of the B. Serrata (Boswellia Serrata Roxb. ex Colebr.) gum resin, with a declared 65% *w*/*w* concentration of boswellic acids was purchased from Farmalabor Srl (Canosa di Puglia, Italy).

### 4.2. Sample Preparation and Epoxidation Reaction Conditions

The powder extract of the B. Serrata gum resin was dissolved in methanol to a nominal concentration of 1.0 mg/mL. The sample was filtered on a 0.2 μm Nylon filter and subsequently diluted by a 1:10 factor in an 80:20 *v*/*v* methanol/water prior to RPLC-ESI-FTMS analysis.

The epoxidation reaction with m-CPBA was performed on an extract of the *B. Serrata* gum resin powder following the protocol proposed by Coniglio et al. [39]. The powder extract was dissolved in pure chloroform to a nominal concentration of 100 μg/mL. A total of 1 mL of the resulting solution was diluted with 1 mL of a 1 mg/mL chloroform solution of m-CPBA. The mixture was kept under continuous stirring for 15 min at room temperature (22 °C). Thereafter, the reaction was quenched after the addition of 2 mL of water. Here, the two-phase system was subjected to vigorous agitation using a vortex mixer. A clear two-phase separation was obtained again after centrifugation (10 min, 4500× *g*). The organic phase was withdrawn and subjected to solvent evaporation under nitrogen flow. The dry residue was redissolved in 1 mL of an 80:20 *v*/*v* methanol/water mixture before RPLC-FTMS analysis.

### 4.3. LC-MS Instrumentation and Operating Conditions

RPLC-ESI(-)-FTMS/MS analyses were performed using an LC-MS platform including an Ultimate 3000 HPLC quaternary chromatographic system and a Q Exactive high-resolution quadrupole-Orbitrap mass spectrometer (Thermo Fisher, West Palm Beach, CA, USA). Here, the chromatographic column effluent was transferred into the heated electrospray ionization (HESI) interface (Thermo Fisher, West Palm Beach, CA, USA) mounted on the mass spectrometer. The RPLC separations of BAs, their isomers, and the products of the epoxidation reaction were performed using a C18 Ascentis Express column (15 cm length, 2.1 mm internal diameter) packed with core–shell 2.7 μm particles (Supelco, Bellefonte, PA, USA) and operated at a 0.2 mL/min flow. A 5 μL sample volume was subjected to RPLC-ESI(-)-FTMS/MS analysis. The separation of BAs and their isomers was obtained using a modified version of the chromatographic approach proposed by Schmiech et al. [7]. The following multistep binary elution gradient, based on an 80:20 *v*/*v* methanol/water mixture as phase A and methanol as phase B, both containing a 2.5 mM nominal concentration of ammonium acetate and ammonia, was adopted: 0–2 min) isocratic at 10% B; 2–13 min) linear increase of B from 10% to 20%; 13–20 min) linear increase of B from 20% to 70%; 20–27 min) isocratic at 70% B; 27–29 min) linear decrease of B from 70% to 10%; 29–40 min) isocratic re-equilibration at 10% B. The parameters of the HESI interface and the ion optics of the Q-Exactive spectrometer were set as follows: sheath gas flow rate) 40 a.u.; auxiliary gas flow rate) 15 a.u.; spray voltage) −3 kV; capillary temperature) 200 °C; S-lens RF level 60. The mass spectrometer was operated at its maximum resolving power (140,000 at *m*/*z* 200) for both full scan MS and MS/MS experiments. Full-scan high-resolution MS spectra were acquired in a 300–700 *m*/*z* interval. Here, the automatic gain control (AGC) level was set to 1 × 10^6^, with a maximum injection time of 100 ms. A normalized collision energy (NCE) of 60 units was adopted for MS/MS experiments, while the AGC level was set to 2 × 10^5^, with a maximum injection time of 100 ms. The spectrometer was calibrated daily by infusing, at a 5 μL/min flow rate, calibration solutions provided by the instrument manufacturer for negative polarity acquisitions. As a result, a mass accuracy always better than 5 ppm was achieved.

### 4.4. Hydrogen/Deuterium Exchange Experiments

Both β-BA and α-BA were subjected to hydrogen/deuterium (H/D) experiments to rationalize the main fragmentation pathways inferred from the ESI(−)-FTMS/MS spectra of the corresponding [M−H]^−^ ions. To such purpose, 500 μL of pure methanol were spiked with 10 μL of a 1 mg/mL methanolic solution of α-BA. The same operation was performed in a different vial for β-BA. The two solutions were dried under nitrogen flow. The dried residues were redissolved in 1 mL of a CH_3_CN/D_2_O 80:20 (*v*/*v*) mixture. Then, 1 μL of pure formic acid was added to catalyze the H/D exchange. The resulting solutions were vigorously stirred for 1 min and kept quiescent for 10 min. Thereafter, the solutions containing the deuterated forms of α-BA and β-BA were directly infused in the ESI source for the acquisition of FTMS/MS spectra pertaining to *m*/*z* 456.4 ions. The same ion source and MS parameters described in Section 4.3 for MS/MS experiments were adopted.

### 4.5. MS/MS Data Processing for Principal Component Analysis

Once exported in .txt format, MS/MS data were processed by using an in-house-developed R-script to determine the chemical formula of each product ion starting from accurate *m*/*z* values. Only signals exhibiting intensities higher than 2% of the base peak were considered. Thereafter, the corresponding theoretical *m*/*z* values were calculated and paired with the measured relative intensities. This information was merged for all boswellic acids isomers using a conservative approach (i.e., product ions that were not common to all MS/MS spectra were also considered and their intensities were considered equal to 0 when they were not detected). Data were arranged into the format required by BiPlotteR (https://ancaste.github.io/BiPlotteR/, accessed on 18 October 2024), i.e., the R-package that was exploited to perform principal component analysis (PCA), based on the covariance matrix. The outcome of the PCA was further processed with Sigmaplot (v.14.0) to obtain a personalized graphical visualization for the score and the loading plots.

## Figures and Tables

**Table 1:** List of boswellic acids (BAs) and their isomers, numbered as in Figure 1, tentatively identified in a commercial sample of lyophilized *Boswellia serrata* extract. Only abbreviated names are shown in the table (see Tables S1 and S2 for detailed information about the chemical structure, trivial and systematic names). The fourth column displays the *m*/*z* values related to the 5 most intense signals, in order of intensity, detected in the corresponding HCD tandem mass spectra, with bold values referring to ions for which structures were hypothesized. Red-colored values in the column refer to diagnostic fragment ions that were not included in the top 5 list. In the fifth column, three reactivity levels are proposed to qualitatively define the yield of epoxidation reaction with *m*-CPBA: high (the EIC peak was not recognized after the reaction), moderate (the EIC peak was still recognizable after the reaction but with a significantly reduced area), and low (the EIC peak area remained substantially unaltered after the epoxidation reaction). Wavelengths related to characteristic absorptions found in UV spectra are reported in the last column.

Identified Species	Peak Number (see the BPC in Figure 1)	*m*/*z* for [M-H]^−^ ion	*m/z* Values for Top 5 MS/MS Peaks and Diagnostic Ions	Reactivity towards Epoxidation with *m*-CPBA	Wavelengths of Distinctive UV Absorption Maxima
β-KBA	1	469.3323	**391.3009**, 407.2592 375.2694, 361.2539 407.3319, **60.9931**	Low	249 nm
β-KBA isomer	2	469.3323	**391.3009**, 407.2592 375.2694, 361.2539 407.3319, **60.9931**	Low	249 nm
β-AKBA	3	511.3429	59.0138	Low	249 nm
OA	4	455.3531	455.3531 407.3325	Moderate	None
UA	5	455.3531	455.3531 407.3325	Low	None
α-7,24-TDA, α-EA, and β-EA	6	455.3531	455.3531, **425.3068** 339.2696, 97.0659 **373.2752**		
	8		455.3531, **425.3069** **373.2753**, 437.343 339.2696	High	None
	9		455.3531, **425.3070** **373.2753**, 339.2696 437.3432		
3-oxo-TA	7	453.3374	453.3377, 97.0658 **423.2909**, 85.0658 **371.2593**	High	None
α-7,24- ATDA, α-AEA, and β-AEA	10	497.3636	**437.3426**, 59.0138 497.3640, 467.3166 **355.2645**, **407.2956**		
	13		**437.3426**, 59.0138 497.3639, 467.3167 419.3324, **355.2645** **407.2956**	High	None
	14		**437.3426**, 59.0138 497.364, 467.3166 419.3323, **355.2645** **407.2956**		
α-DHBA and β-DHBA	11	453.3374	73.0294, **375.3058** **359.2746**, 407.2957 **60.9931**	Moderate	281 nm
	15		73.0295, **359.2747** **375.3059**, **60.9931** 407.2957		
LA	12	455.3531	**437.3430**, **409.3482** **407.3326**, **60.9932** 455.3538	High	None
α-BA	16	455.3531	**437.3430, 409.3482** **60.9932**, 455.3538 **407.2961**, **407.3325** **409.3118**, **219.1392**	Moderate	None
β-BA	17	455.3531	**437.3431**, **409.3118** **409.3482**, 391.3013 **60.9932**, **407.2961** **407.3325**, **219.1392**	Low	None
α-ADHBA and β-ADHBA	18	495.3480	**59.0138**	Moderate	281 nm
	21		**59.0138**		
ALA	19	497.3636	**59.0138**	High	None
epi-β-BA	20	455.3531	**437.3431**, 455.3538 **409.3118**, **409.3482** **60.9932**, **407.3325** **407.2961**, **219.1393**	Low	None
α-ABA	22	497.3636	**59.0138**	Moderate	None
β-ABA	23	497.3636	**59.0138**	Low	None

## Data Availability

The data presented in this study are available on request from the corresponding authors.

## Supplementary Materials

The following supporting information can be downloaded at: https://www.mdpi.com/article/10.3390/molecules29204967/s1, Table S1: Summary of chemical information on BAs identified during this study or reported in the literature for *B. serrata*; Table S2: Summary of chemical information on BA isomers identified during this study or reported in the literature for *B. serrata*; Figure S1: ESI(−)-FTMS/MS spectra for α-BA and β-BA standards and for peaks b_7_ and b_6_; Figure S2: ESI(−)-FTMS/MS spectra for peaks b_1_–b_5_; Figure S3: ESI(−)-FTMS/MS spectra for peaks a_1_–a_6_; Figure S4: EIC traces for ketoboswellic acids and UV chromatograms acquired at 249 and 281 nm; Figure S5: ESI(−)-FTMS/MS spectra for peaks k_1_, d_1_ and d_3_; Scheme S1: Fragmentation pathways involving the A and B ring of α-BA and β-BA [M-H]^−^ ions; Scheme S2: Fragmentations pathways proposed to explain the detection of product ions compatible with exact *m/z* values 409.3112 and 407.2956; Scheme S3: Fragmentation processes proposed for structural features that are common among BAs and 3-hydroxytirucallic acid isomers; Scheme S4: Fragmentation mechanisms proposed to explain the main signals observed in the ESI(−)-FTMS/MS spectra of acetylated LA, α-BA, β-BA, and α-7,24-TDA, α-EA, and β-EA; Scheme S5: Fragmentation pathways proposed to explain some of the main signals observed in the ESI(−)-FTMS/MS spectra of nonacetylated ketoboswellic and dehydroboswellic acids.
