# Supplementary material for: Integrating Epoxidation, High-Resolution Mass Spectrometry and Ultraviolet Spectroscopy to Unravel the Complex Profile of Boswellic Acids and Related Compounds in the Boswellia serrata Gum Resin Extract
Source: Molecules. 2024 Oct 21;29(20):4967. doi: 10.3390/molecules29204967 (PMC11510512; doi:10.3390/molecules29204967)
Supplement: Supplementary file 1 [file molecules-29-04967-s001.zip › Losito_Supplementary Material.pdf]

Supplementary Material

Integrating Epoxidation, High-Resolution Mass Spectrometry and Ultraviolet Spectroscopy to Unravel the Complex Profile of Boswellic Acids and Related Compounds in the *Boswellia serrata* Gum Resin Extract

Andrea Castellaneta [1], Ilario Losito [1,2,*], Stefania Cometa [3], Francesco Busto [1], Elvira De Giglio [1,2,4,*] and Tommaso R. I. Cataldi [1,2]

[1] Dipartimento di Chimica, Università degli Studi di Bari "Aldo Moro", via Orabona 4, 70126 Bari, Italy; andrea.castellaneta@uniba.it (A.C.); francesco.busto@uniba.it (F.B.); tommaso.cataldi@uniba.it (T.R.I.C.)
[2] Centro Interdipartimentale SMART, Università degli Studi di Bari "Aldo Moro", via Orabona 4, 70126 Bari, Italy
[3] Jaber Innovation s.r.l., Via Calcutta 8, 00144 Rome, Italy; stefania.cometa@jaber.it
[4] Consorzio Interuniversitario Nazionale per la Scienza e Tecnologia dei Materiali, via Giuseppe Giusti, 9, 50121 Florence, Italy
* Correspondence: ilario.losito@uniba.it (I.L.); elvira.degiglio@uniba.it (E.D.G.)

Table of contents

Table S1. List of the chemical structures, chemical formulas, and the proposed trivial/systematic names for the boswellic acids (BA) that were identified in the present study as well as in other literature studies referring to the chemical composition of *Boswellia serrata* gum resin (page S3).

Table S2. List of the chemical structures, chemical formulas, and the proposed trivial/systematic names for boswellic acids isomers that were identified in the present study as well as in other literature studies referring to the chemical composition of *Boswellia serrata* gum resin (page S8).

Figure S1. ESI(−)-FTMS/MS spectra acquired for the $[M-H]^-$ ions of α-BA and β-BA standards and ESI(−)-FTMS/MS spectra averaged under peaks b_7 and b_6 detected in the EIC trace reported in Figure 2A (*m/z* 455.3531) (page S12).

Figure S2. ESI(−)-FTMS/MS spectra averaged under peaks b_1, b_2, b_3, b_4, and b_5 detected in the EIC trace reported in Figure 2A (*m/z* 455.3531) (page S13).

Figure S3. ESI(−)-FTMS/MS spectra averaged under peaks a_4, a_5, a_6, a_1, a_2, and a_3, detected in the EIC trace reported in Figure 2C (*m/z* 497.3636) (page S14).

Figure S4. Comparisons between EIC traces obtained by RPLC-ESI(−)-FTMS for $[M-H]^-$ ions of β-ketoboswellic (*m/z* 469.3323) and acetylated β-ketoboswellic (*m/z* 511.3429) acids, and of dehydroboswellic (*m/z* 453.3374) and acetylated dehydroboswellic (*m/z* 495.3480) acids. RPLC-UV-DAD chromatograms acquired at 249 nm and 281 nm (page S15).

Figure S5. ESI(−)-FTMS/MS spectra averaged under specific peaks detected in the RPLC-ESI(−)-FTMS traces obtained for *m/z* 469.3323 (peak k_1) and *m/z* 453.3374 (peaks d_1 and d_3) ions (page S16).

Scheme S1. Fragmentation pathways involving the A and B ring of α-BA and β-BA $[M-H]^-$ ions proposed to explain the presence of some of the peak signals detected in the ESI(−)-FTMS/MS spectra of mono-deuterated $[M-H]^-$ ions (page S17).

Scheme S2. Fragmentations pathways proposed to explain the detection of product ions compatible with exact *m/z* values 409.3112 and 407.2956 in the ESI(−)-FTMS/MS spectra of α-BA (A) and β-BA (B) $[M-H]^-$ ions (page S18).

Scheme S3. Fragmentation processes proposed for structural features that are common among BAs and 3-hydroxytirucallic acid isomers (page S19).

Scheme S4. Fragmentation mechanisms proposed to explain the main signals observed in the ESI(−)-FTMS/MS spectra of acetylated LA, α-BA, β-BA, and α-7,24-TDA, α-EA, and β-EA (page S20).

Scheme S5. Fragmentation pathways proposed to explain some of the main signals observed in the ESI(−)-FTMS/MS of non-acetylated ketoboswellic and dehydroboswellic acids (page S21).

Supplementary tables

Table S1. List of the chemical structures, chemical formulas, and the proposed trivial/systematic names for the boswellic acids (BA) that were identified in the present study as well as in other literature studies referring to the chemical composition of *Boswellia serrata* gum resin (see Refs. 8 and 17 in the main text). The molecules were classified in two main groups, *i.e.*, oleanane-type (O-type) BA and ursane-type (U-type) BA. α-boswellic acid (α-BA) and its derivatives belong to the O-type group, since they share the same oleanane backbone. On the other hand, the U-type group comprises the β-boswellic acid (β-BA) and its derivatives, *i.e.*, all the BA molecules sharing the same ursane scaffold. Here, the oleanane and ursane backbones, along with the corresponding carbon numbering, were highlighted in red for the chemical structures of α-BA and β-BA, respectively. The table also shows the abbreviations that were used in the main text to address the different BA molecules and the *m/z* value pertaining to the corresponding $[M-H]^-$ ions.

**Chemical structure**	**Chemical formula**	**$[M-H]^-$ *m/z***	**Common name**	**Systematic name**	**Abbreviation**
Oleanane-type (O-type) Boswellic acids					
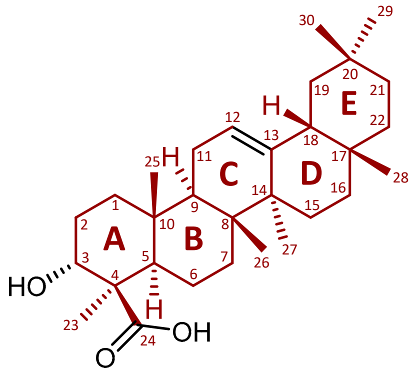	$C_{30}H_{48}O_3$	455.3531	α-boswellic acid	(3α)-hydroxyolean-12-en-24-oic acid	α-BA

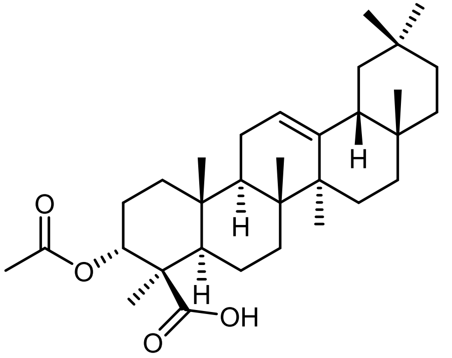	$C_{32}H_{50}O_4$	497.3636	3-acetyl α-boswellic acid	(3α)-O-acetyl olean-12-en-24-oic acid	α-ABA
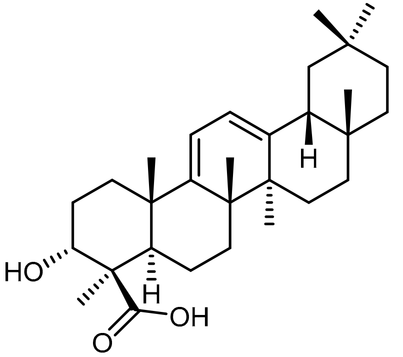	$C_{30}H_{46}O_3$	453.3374	9,11-dehydro-α-boswellic acid	(3α)-hydroxy-9,11-dehydroolean-12-en-24-oic acid	α-DHBA
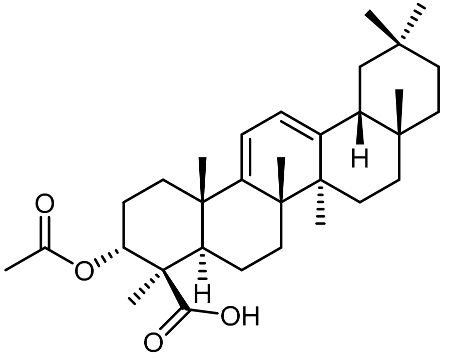	$C_{32}H_{48}O_4$	495.3480	3-acetyl 9,11-dehydro-α-boswellic acid	(3α)-O-acetyl 9,11-dehydroolean-12-en-24-oic acid	α-ADHBA

Ursane-type (U-type) Boswellic acids					
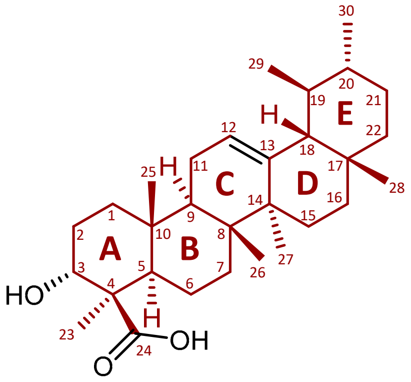	$C_{30}H_{48}O_3$	455.3531	β-boswellic acid	(3α)-hydroxyursan-12-en-24-oic acid	β-BA
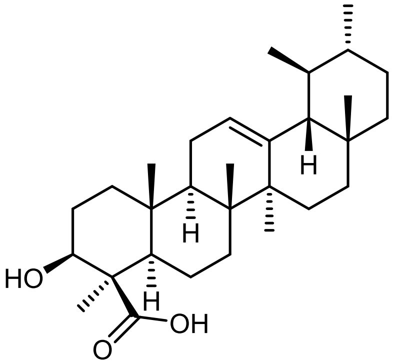	$C_{30}H_{48}O_3$	455.3531	*epi*-β-boswellic acid	(3β)-hydroxyursan-12-en-24-oic acid	*epi*-β-BA
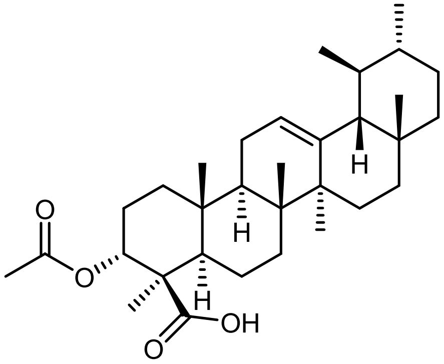	$C_{32}H_{50}O_4$	497.3636	3-acetyl β-boswellic acid	(3α)-O-acetyl ursan-12-en-24-oic acid	β-ABA

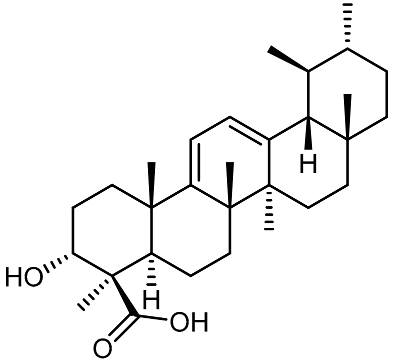	$C_{30}H_{46}O_3$	453.3374	9,11-dehydro-β-boswellic acid	(3α)-hydroxy-9,11-dehydroursan-12-en-24-oic acid	β-DHBA
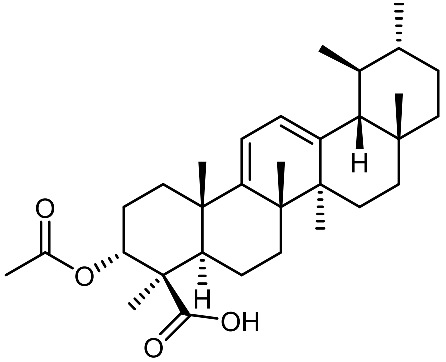	$C_{32}H_{48}O_4$	495.3480	3-acetyl 9,11-dehydro-β-boswellic acid	(3α)-O-acetyl 9,11-dehydroursan-12-en-24-oic acid	β-ADHBA
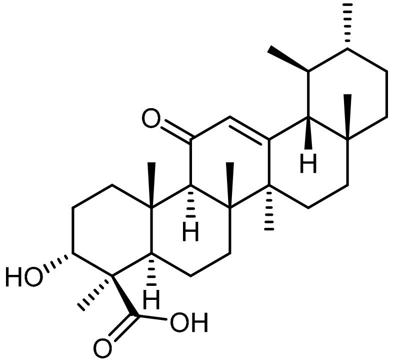	$C_{30}H_{46}O_4$	469.3323	11-keto-β-boswellic acid	(3α)-hydroxy-11-oxours-12-en-24-oic acid	β-KBA

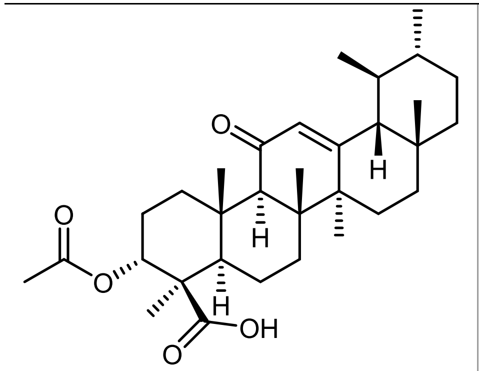	$C_{32}H_{48}O_5$	511.3429	3-acetyl 11-keto-β-boswellic acid	(3α)-O-acetyl 11-oxours-12-en-24-oic acid	β-AKBA

Table S2. List of the chemical structures, chemical formulas, and the proposed trivial/systematic names for boswellic acids isomers that were identified in the present study as well as in other literature studies referring to the chemical composition of *Boswellia serrata* gum resin (see Refs. 8 and 17 in the main text). The table also includes the *m/z* values of the corresponding $[M\text{-}H]^-$ ions.

Chemical structure	Chemical formula	$[M\text{-}H]^-$ *m/z*	Common name	Systematic name	Abbreviation
α-BA and β-BA isomers					
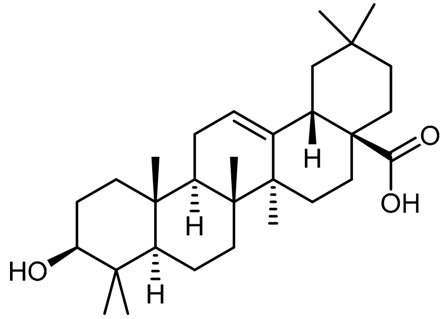	$C_{30}H_{48}O_3$	455.3531	Oleanolic acid	(3β)-hydroxyolean-12-en-28-oic acid	OA
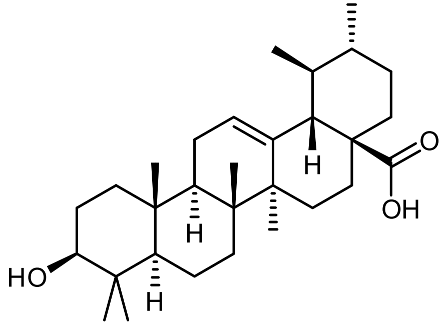	$C_{30}H_{48}O_3$	455.3531	Ursolic Acid	(3β)-hydroxyurs-12-en-28-oic acid	UA

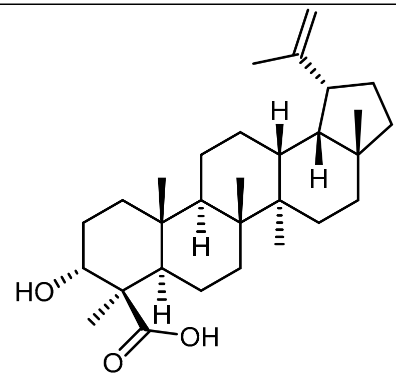	$C_{30}H_{48}O_3$	455.3531	Lupeolic acid	(3α)-hydroxylup-20(29)-en-24-oic acid	LA
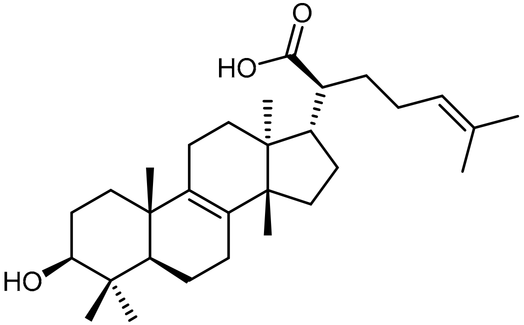	$C_{30}H_{48}O_3$	455.3531	β-elemolic acid	(3β)-hydroxytirucalla-8,24-dien-21-oic acid	β-EA
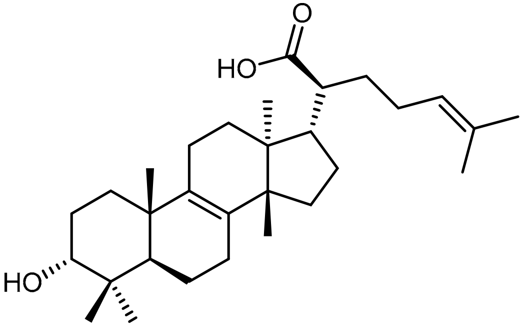	$C_{30}H_{48}O_3$	455.3531	α-elemolic acid	(3α)-hydroxytirucalla-8,24-dien-21-oic acid	α-EA

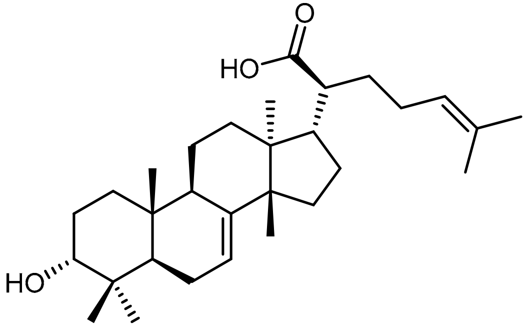	$C_{30}H_{48}O_3$	455.3531	-	(3α)-hydroxytirucalla-7,24-dien-21-oic acid	α-7,24-TDA
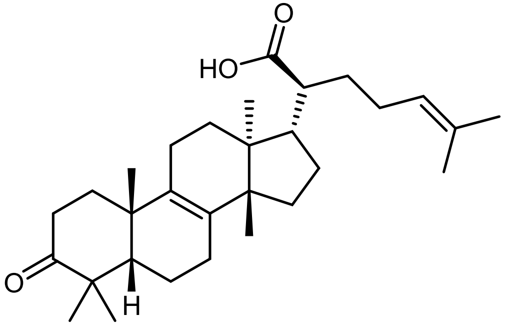	$C_{30}H_{46}O_3$	453.3374	3-oxo-tirucallic acid	3-oxo-tirucalla-8,24-dien-21-oic acid	3-oxo-TA
α-ABA and β-ABA isomers					
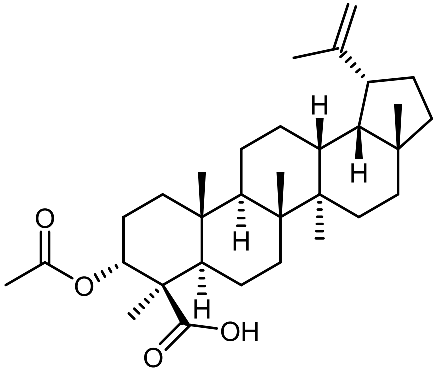	$C_{32}H_{50}O_4$	497.3636	3-acetyl lupeolic acid	(3α)-O-acetyl lup-20(29)-en-24-oic acid	3

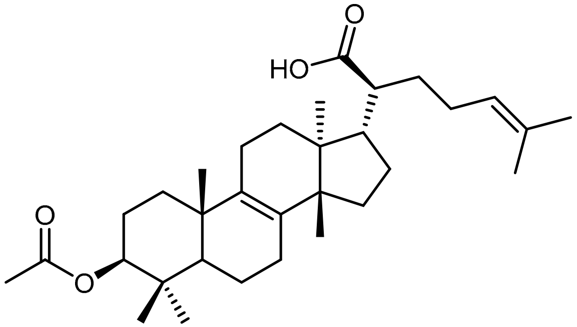	$C_{32}H_{50}O_4$	497.3636	-	(3β)-O-acetyl tir-8,24-dien-21-oic acid	β-AEA
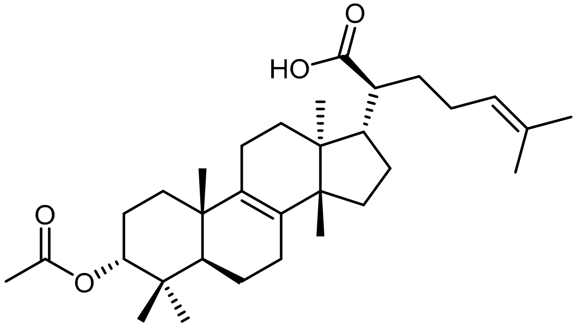	$C_{32}H_{50}O_4$	497.3636		(3α)-O-acetyl tir-8,24-dien-21-oic acid	α-AEA
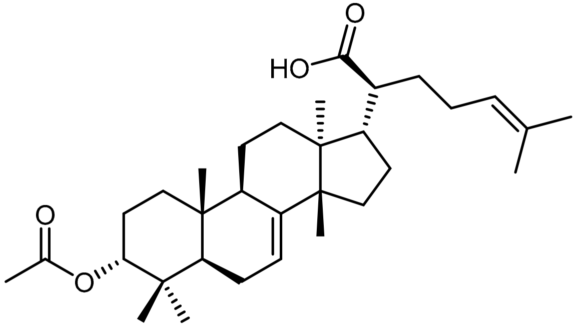	$C_{32}H_{50}O_4$	497.3636		(3α)-O-acetyl tir-7,24-dien-21-oic acid	α-7,24-ATDA

Supplementary figures

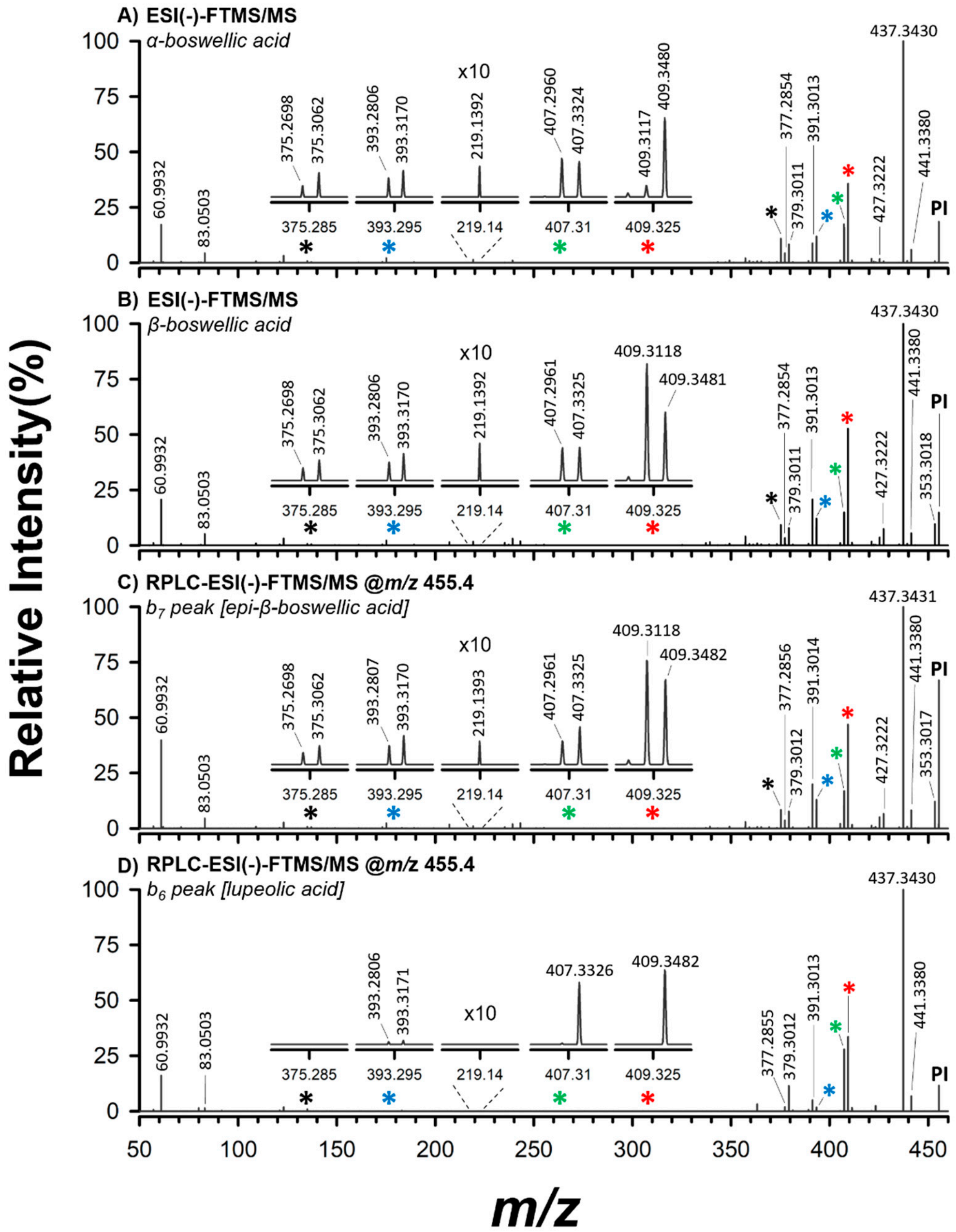

Figure S1. Panels A and B show the ESI(−)-FTMS/MS spectra acquired for the [M−H]⁻ ions of α-BA and β-BA standards, respectively. Panels C and D display the averaged ESI(−)-FTMS/MS spectra for peaks b_7 (panel C) and b_6 (panel D) detected in the EIC trace reported in Figure 2A, tentatively identified as *epi*-β-BA and LA, respectively (see Table S1, Table S2, and the main text for details). In all the spectra, the precursor ion peak (*m/z* 455.4) was marked as "PI". Each panel also includes a magnified view of the spectral regions marked by colored asterisks.

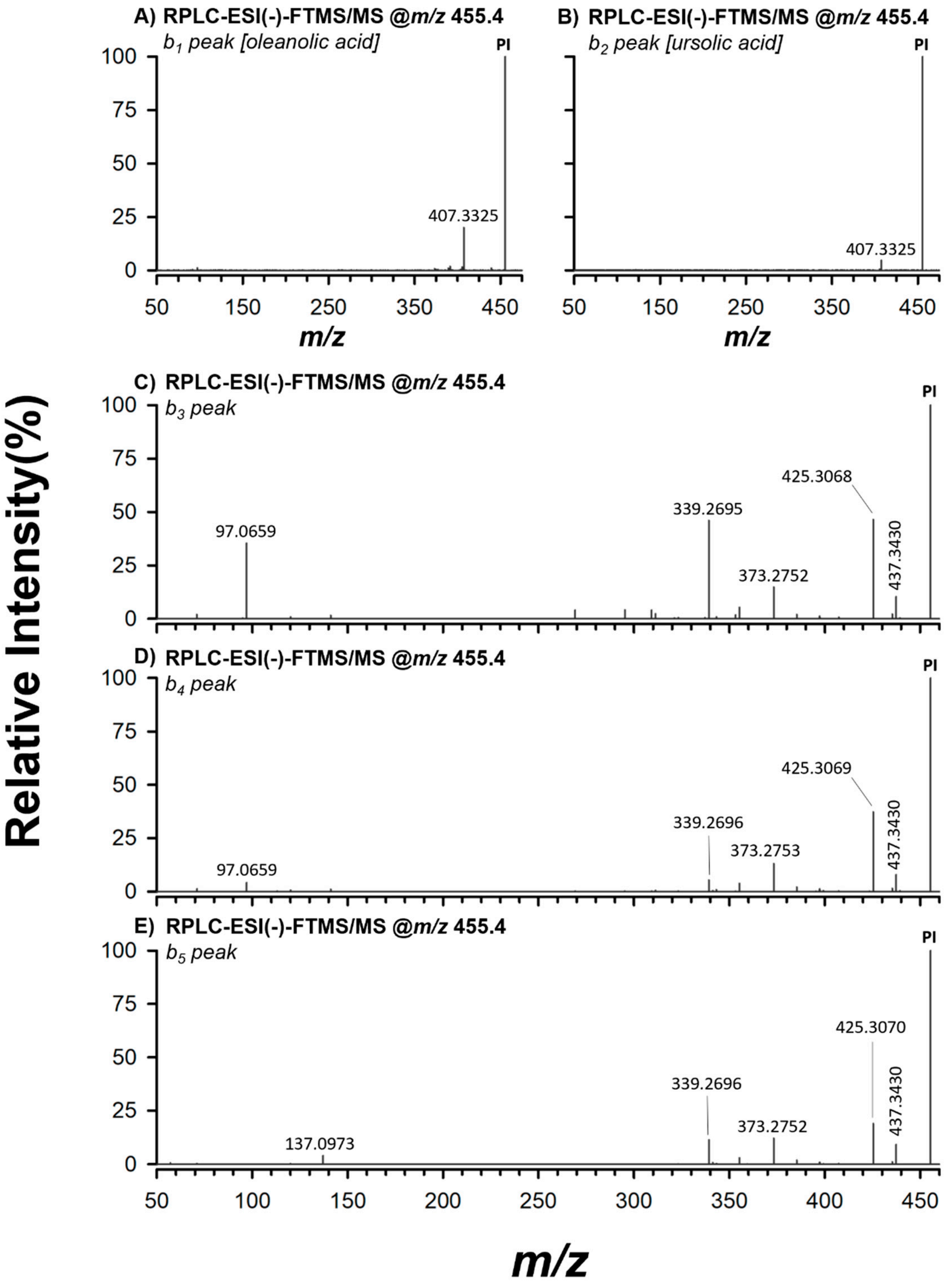

Figure S2. ESI(-)-FTMS/MS spectra averaged under specific peaks detected in the RPLC-ESI(−)-FTMS EIC trace obtained for *m/z* 455.3531 ions and reported in Figure 2A: b_1 (panel A), b_2 (panel B), b_3 (panel C), b_4 (panel D), and b_5 (panel E). The peaks were tentatively attributed to OA, UA, and α-7,24-TDA, α-EA, β-EA, respectively (see Table S1, Table S2, and the main text for details). In all the spectra, the precursor ion peak (*m/z* 455.4) was labelled as “PI”.

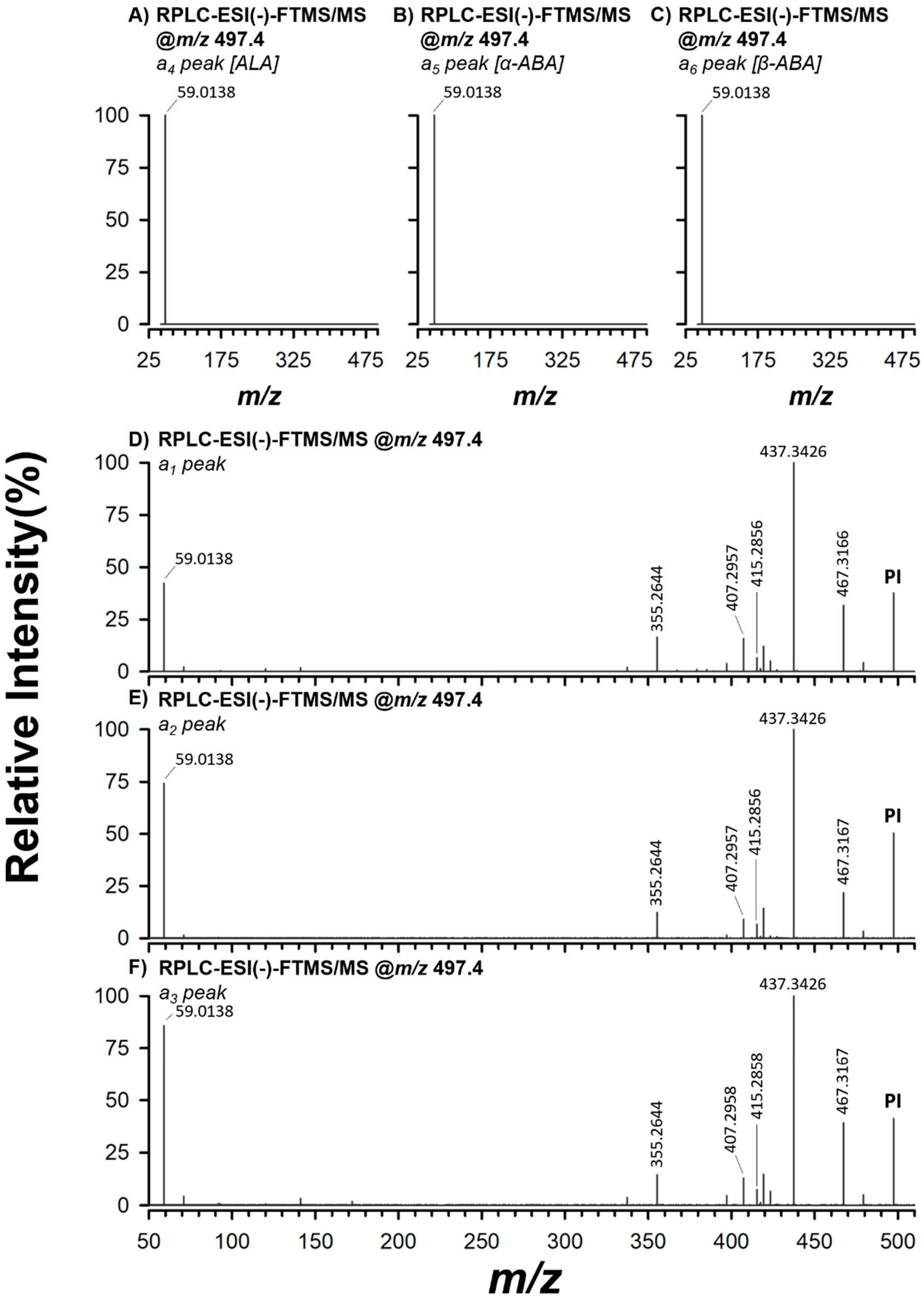

Figure S3. ESI(-)-FTMS/MS spectra averaged under specific peaks of the RPLC-ESI(−)-FTMS EIC trace obtained for *m/z* 497.3636 ions (see Figure 2C): a_4 (A), a_5 (B), a_6 (C), a_1 (D), a_2 (E), and a_3 (F). The peaks were tentatively identified as the acetylated forms of LA, α-BA, β-BA OA, and α-7,24-TDA, α-EA, β-EA, respectively (see Table S1, Table S2, and the main text for details). In all spectra, the precursor ion peak (*m/z* 497.4) was labelled as “PI”.

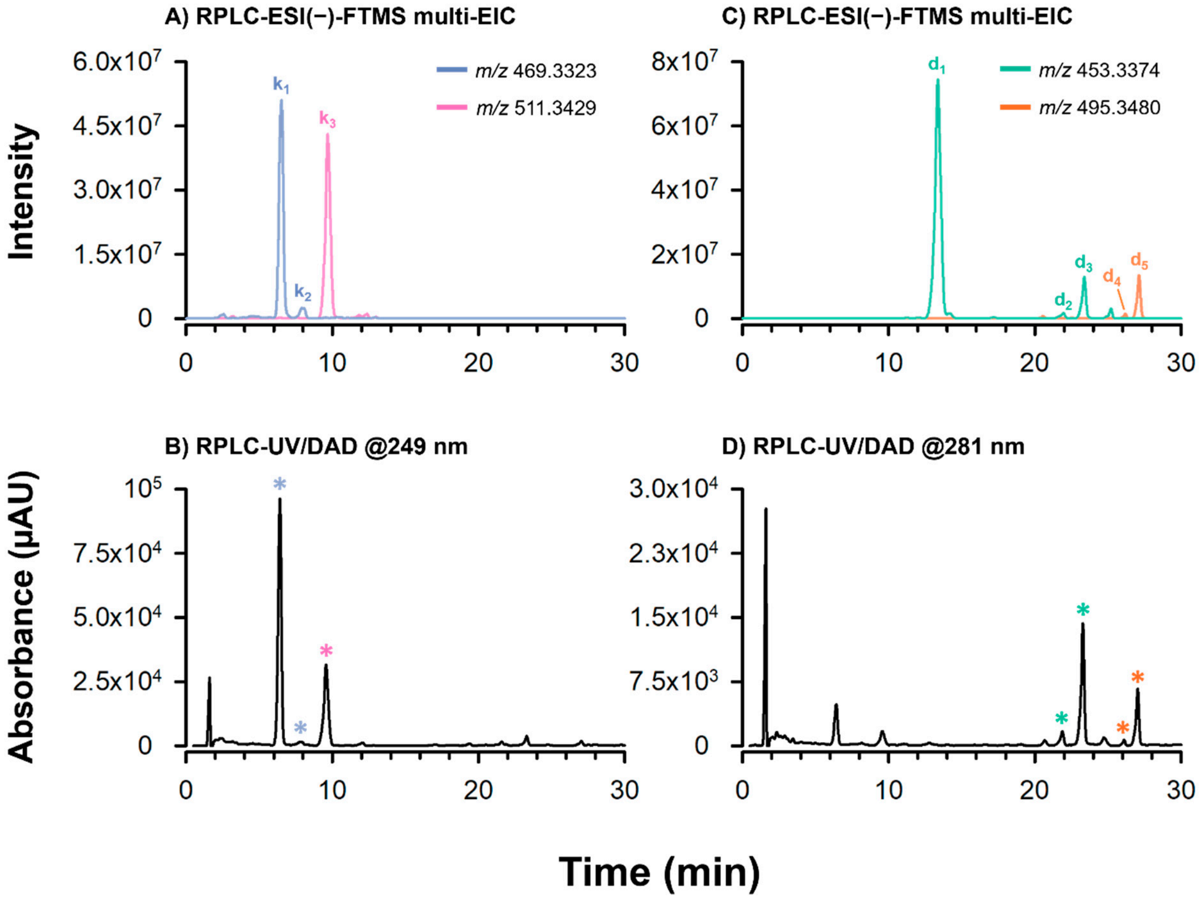

Figure S4. A) EIC traces obtained by RPLC-ESI(−)-FTMS for [M−H]⁻ ions of known ketoboswellic acids, namely β-KBA (*m/z* 469.3323) and β-AKBA (*m/z* 511.3429). C) EIC traces for [M−H]⁻ ions of dehydroboswellic (*m/z* 453.3374) and acetylated dehydroboswellic acids (*m/z* 495.3480). B and D) RPLC-UV-DAD chromatograms acquired at 249 nm and 281 nm, respectively. The asterisk-labelled peaks in the RPLC-UV traces were unambiguously aligned with peaks in the EIC chromatograms. The peak alignment is highlighted by the adoption of the same colour code for asterisks and EIC traces.

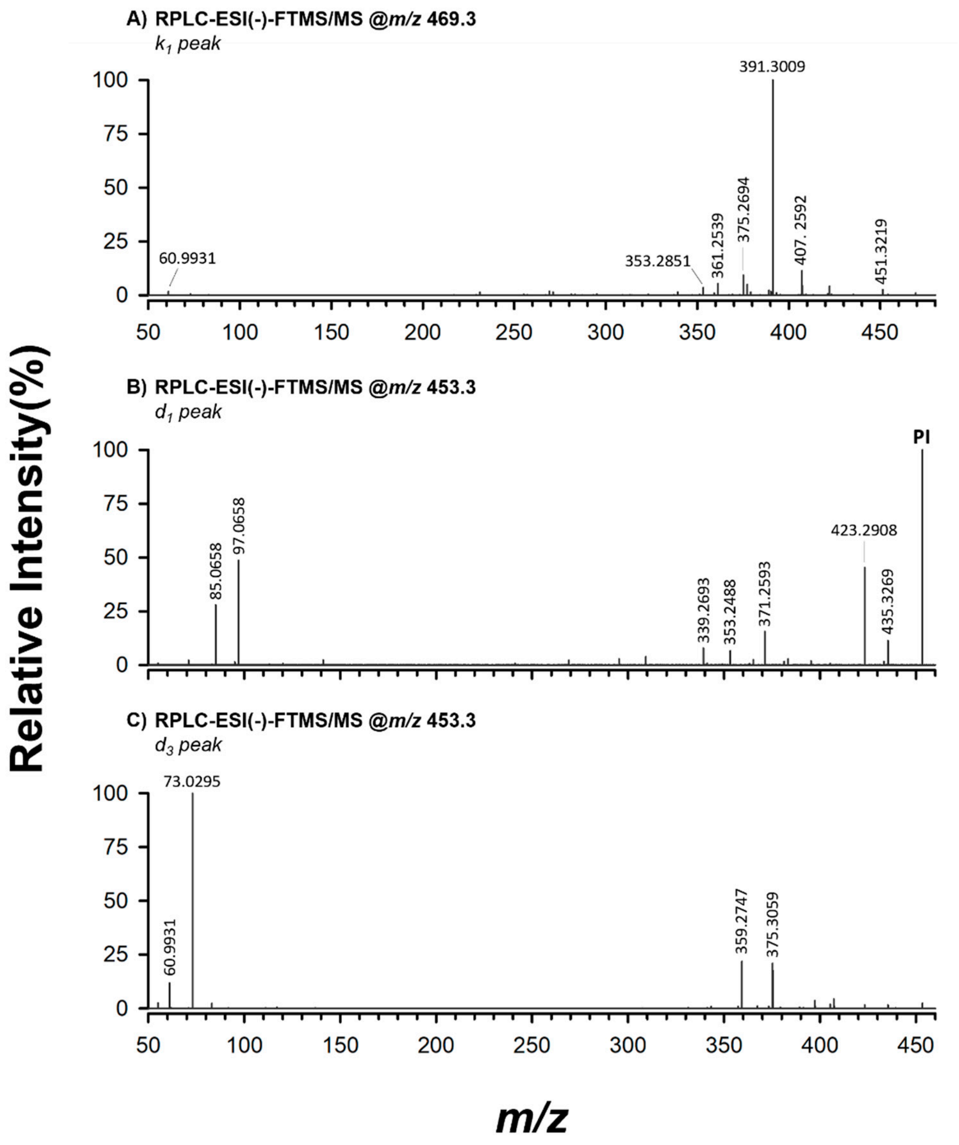

Figure S5. ESI(-)-FTMS/MS spectra corresponding to specific peaks detected in the RPLC-ESI(−)-FTMS traces obtained for *m/z* 469.3323 (A) and *m/z* 453.3374 (B and C) ions shown in Figures S4A and S4B: k_1 (A), d_1 (B), and d_3 (C). The peaks were tentatively identified as β-KBA, 3-oxo-TA, and β-DHBA, respectively (see Table S1). If detected, the precursor ion peak was labelled as "PI".

Supplementary schemes

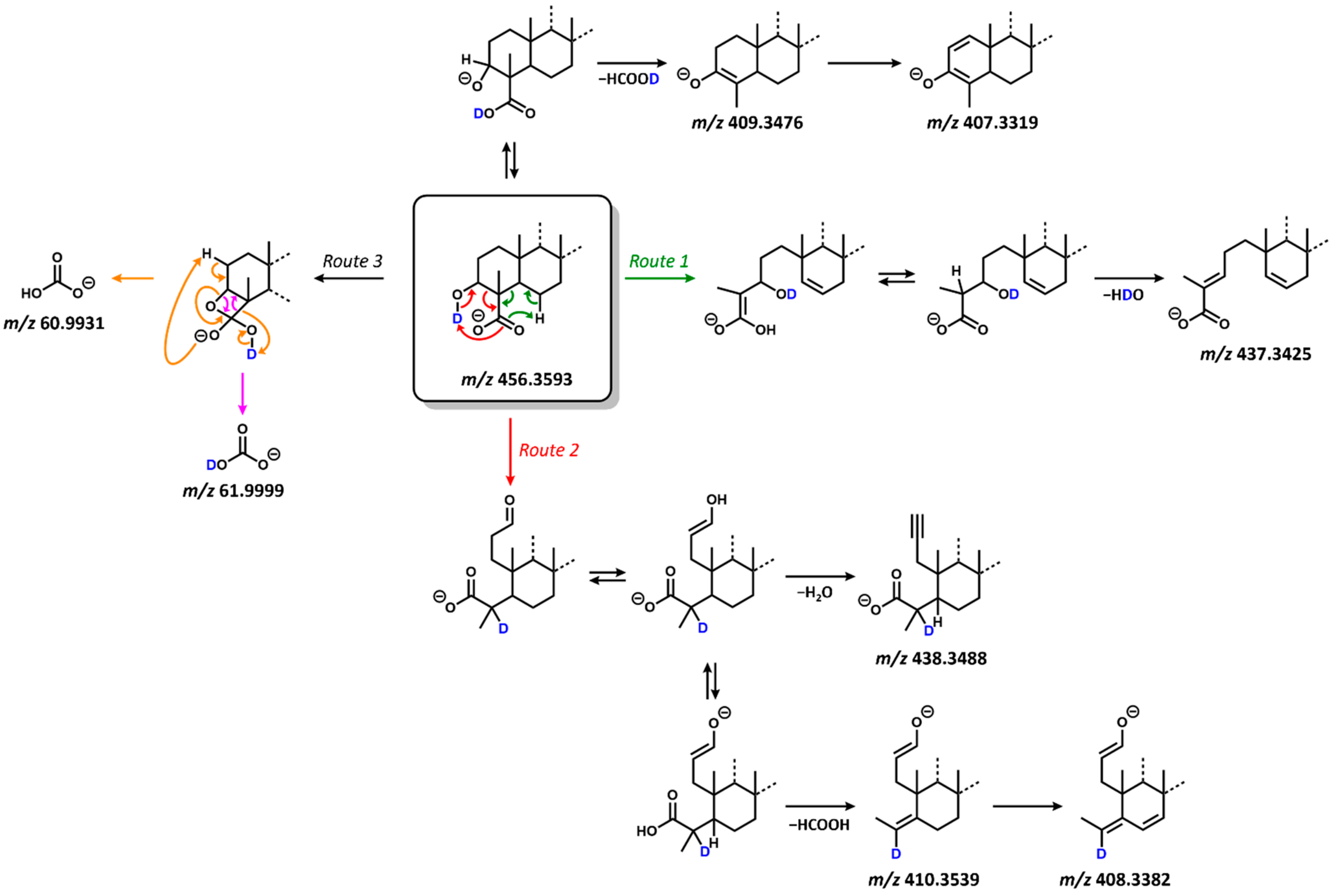

Scheme S1. Fragmentation pathways involving the A and B ring of α-BA and β-BA [M-H]⁻ ions proposed to explain the presence of some of the peak signals detected in the ESI(−)-FTMS/MS spectra of mono-deuterated [M-H]⁻ ions (see Figure 4). For the sake of simplicity, only those parts of the chemical structures that were assumed to be actively involved in the fragmentation process are depicted.

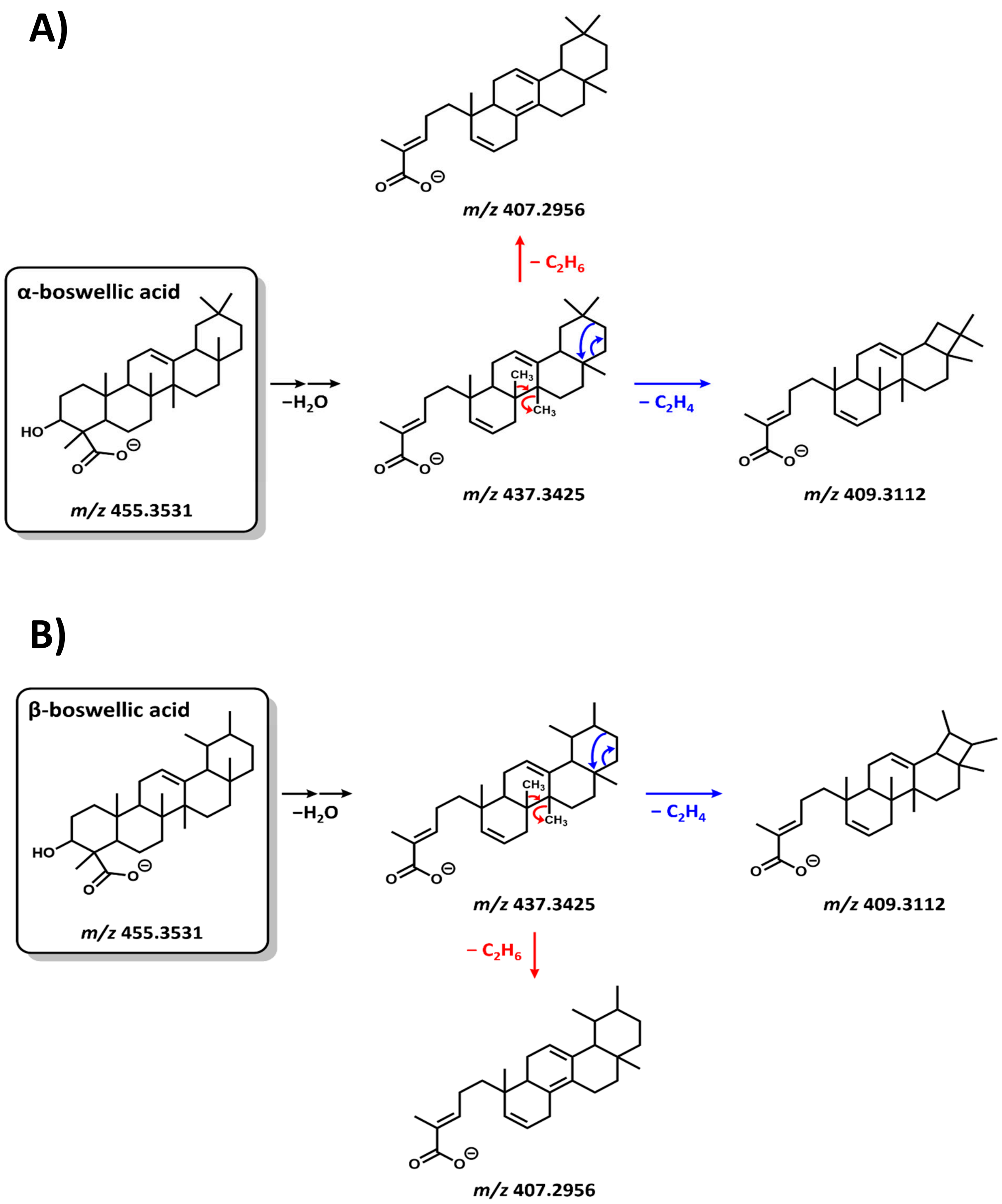

Scheme S2. Fragmentations pathways proposed to explain the detection of product ions compatible with exact *m/z* values 409.3112 and 407.2956 in the ESI(−)-FTMS/MS spectra of α-BA (A) and β-BA (B) $[M\text{-}H]^-$ ions. The same product ions were generated by the collision induced dissociation of *epi*-β-BA $[M\text{-}H]^-$ ions, but not in the case of deprotonated LA molecules (see Figure S1).

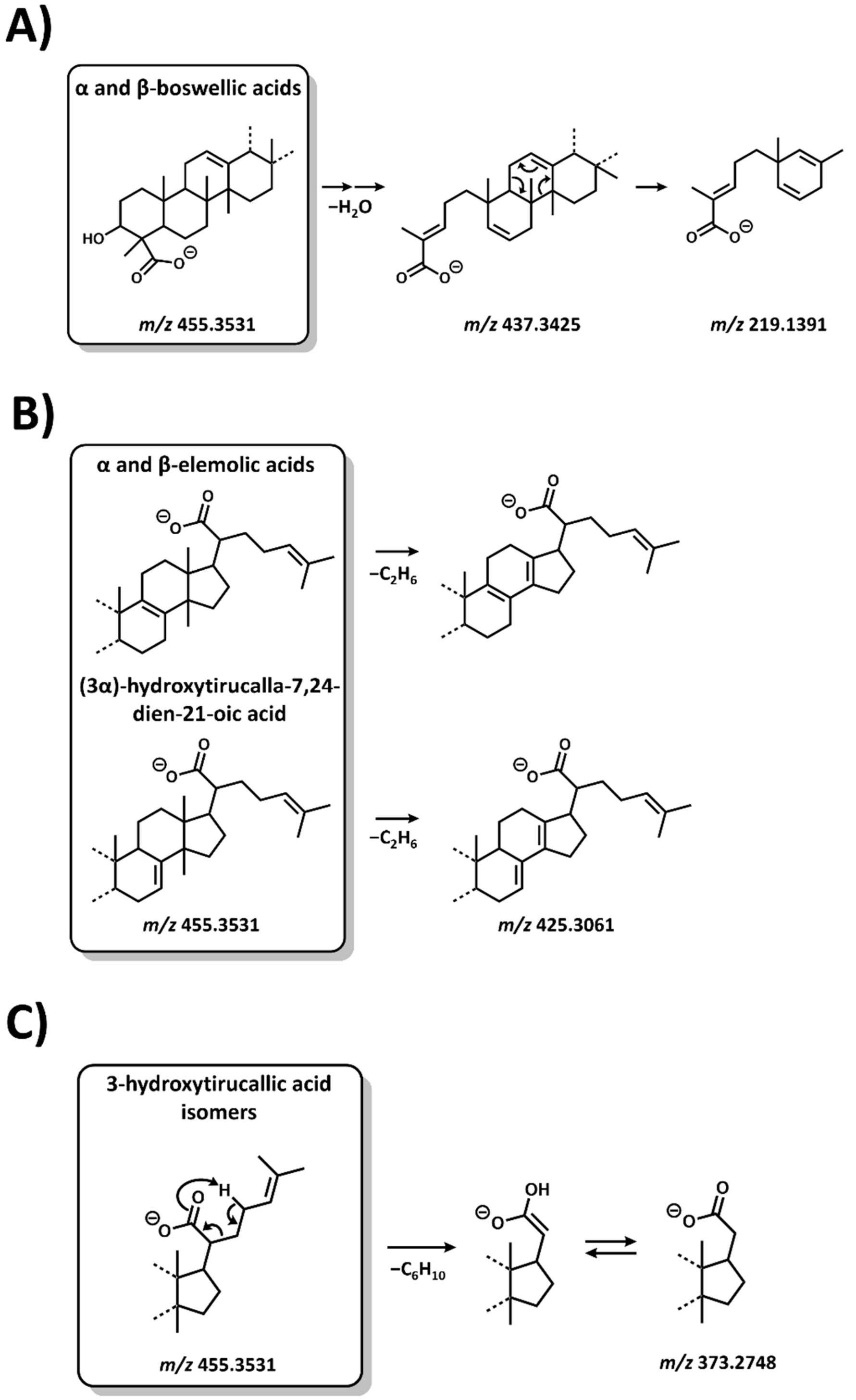

Scheme S3. Fragmentation processes proposed for structural features that are common among BAs (**A**) and 3-hydroxytirucallic acid isomers (**B** and **C**). In the MS/MS spectra of BA $[M-H]^-$ ions, the formation of the *m/*z 219.1391 ion was attributed to a retro-Diels Alder reaction involving the double bond between C12 and C13 (**A**). The extended conjugation of the double bond may explain the formation of the ion at *m/z* 425.3061 in the case of α-EA, β-EA, and α-7,24-TDA (**B**). A McLafferty-like rearrangement (panel **C**) was invoked by Katragunta *et al*. (see Ref. 4) to explain the formation of *m/z* 373.2748 in the MS/MS spectra of the corresponding $[M-H]^-$ ions. For the sake of simplicity, the figure display only the portion of the chemical structures believed to be involved in the fragmentation process.

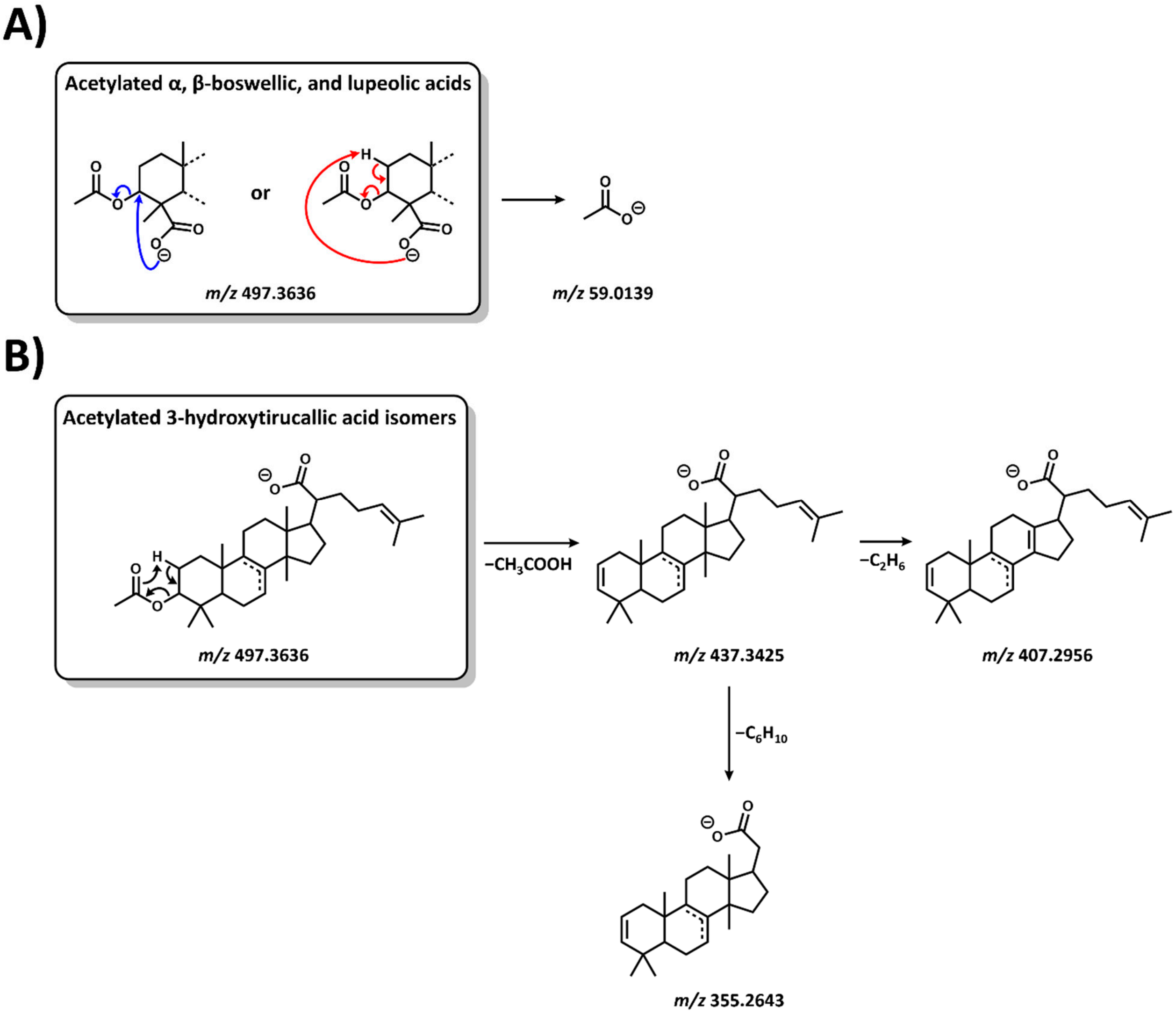

Scheme S4. Fragmentation mechanisms proposed to explain the main signals observed in the ESI(−)-FTMS/MS spectra of acetylated LA, α-BA, β-BA, and α-7,24-TDA, α-EA, and β-EA. A) For ALA, α-ABA, and β-ABA, the presence of a carboxylic group in vicinal position to the acetylated hydroxylic group determined the dominant detachment of an acetate ion either by a direct nucleophilic attack (blue arrows) or through a β-elimination mechanism (red arrows). B) for 7,24-ATDA, α-AEA, and β-AEA the absence of a vicinal nucleophile opened the way to side fragmentation mechanisms that were analogously observed for the corresponding non-acetylated forms (B). For the sake of simplicity, panel A shows only those parts of the chemical structures that were assumed to be actively involved in the fragmentation process.

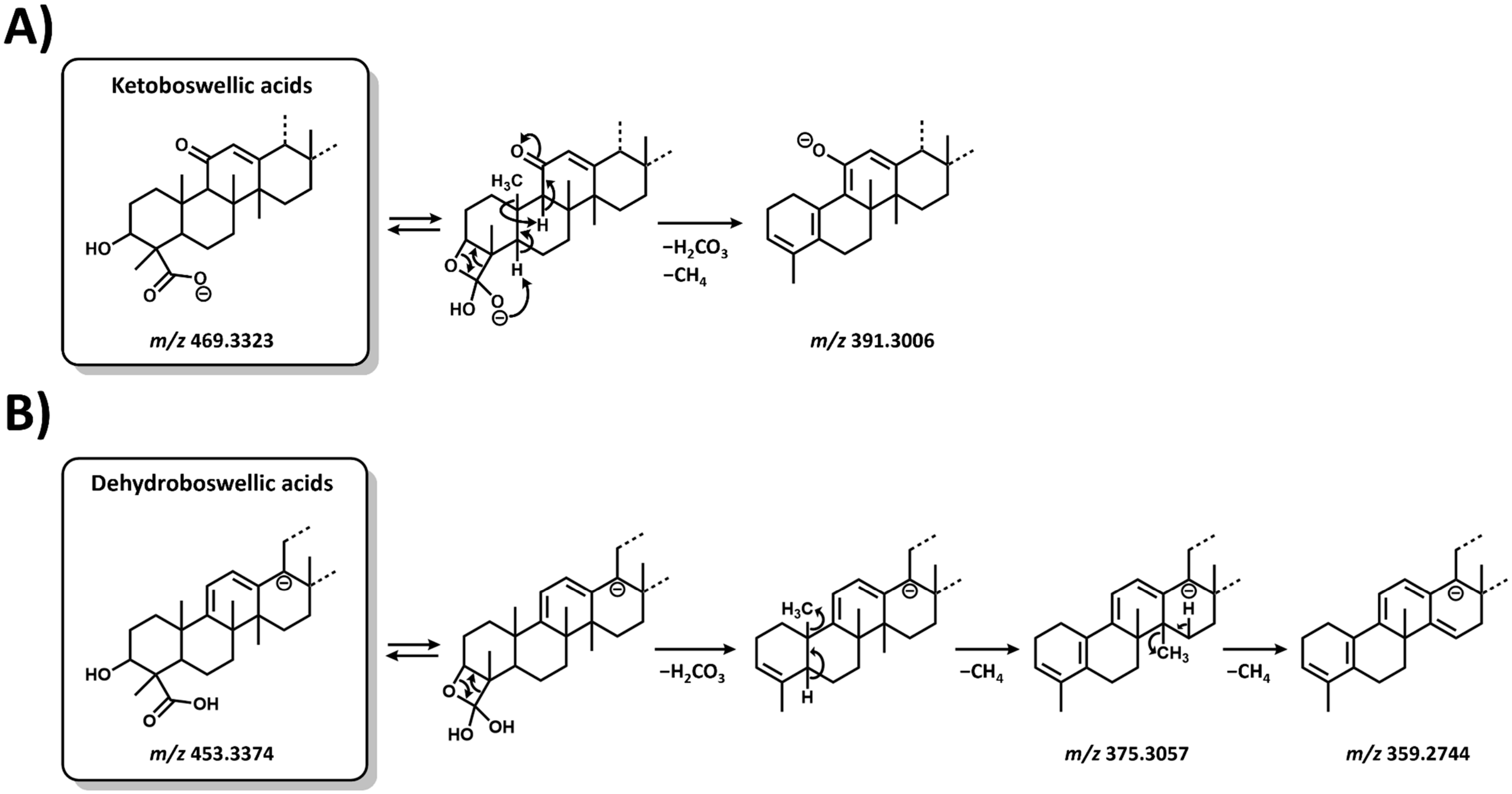

Scheme S5. Fragmentation pathways proposed to explain some of the main signals observed in the ESI(−)-FTMS/MS of non-acetylated ketoboswellic and dehydroboswellic acids. For the sake of simplicity, the figure shows only those parts of the chemical structures that were assumed to be actively involved in the fragmentation process.
